# The LIN28B–let‐7–PBK pathway is essential for group 3 medulloblastoma tumor growth and survival

**DOI:** 10.1002/1878-0261.13477

**Published:** 2023-08-07

**Authors:** Shubin W. Shahab, Christianna M. Roggeveen, Jiarong Sun, Haritha Kunhiraman, Leon F. McSwain, Kyle Juraschka, Sachin A. Kumar, Olivier Saulnier, Michael D. Taylor, Matthew Schniederjan, Robert W. Schnepp, Tobey J MacDonald, Anna Marie Kenney

**Affiliations:** ^1^ Aflac Cancer and Blood Disorders Center Children's Healthcare of Atlanta GA USA; ^2^ Department of Pediatrics Emory University School of Medicine Atlanta GA USA; ^3^ Emory College of Arts and Sciences Emory University Atlanta GA USA; ^4^ Department of Neurosurgery, The Hospital for Sick Children University of Toronto ON Canada; ^5^ Department of Laboratory Medicine and Pathology University of Toronto ON Canada; ^6^ The Arthur and Sonia Labatt Brain Tumor Research Centre, The Hospital for Sick Children University of Toronto ON Canada; ^7^ Developmental and Stem Cell Biology Program, The Hospital for Sick Children University of Toronto ON Canada; ^8^ The Janssen Pharmaceutical Ambler PA USA; ^9^ Winship Cancer Institute Atlanta GA USA

**Keywords:** 1632, let‐7, HI‐TOPK‐032, Group 3 medulloblastoma, LIN28B, PBK

## Abstract

Children with Group 3 medulloblastoma (G3 MB) have a very poor prognosis, and many do not survive beyond 5 years after diagnosis. A factor that may contribute to this is the lack of available targeted therapy. Expression of protein lin‐28 homolog B (*LIN28B*), a regulator of developmental timing, is upregulated in several cancers, including G3 MB, and is associated with worse survival in this disease. Here, we investigate the role of the LIN28B pathway in G3 MB and demonstrate that the LIN28B–lethal‐7 (let‐7; a microRNA that is a tumor suppressor)–lymphokine‐activated killer T‐cell‐originated protein kinase (PBK; also known as PDZ‐binding kinase) axis promotes G3 MB proliferation. *LIN28B* knockdown in G3‐MB‐patient‐derived cell lines leads to a significant reduction in cell viability and proliferation *in vitro* and in prolonged survival of mice with orthotopic tumors. The LIN28 inhibitor *N*‐methyl‐*N*‐[3‐(3‐methyl‐1,2,4‐triazolo[4,3‐b]pyridazin‐6‐yl)phenyl]acetamide (1632) significantly reduces G3 MB cell growth and demonstrates efficacy in reducing tumor growth in mouse xenograft models. Inhibiting PBK using HI‐TOPK‐032 also results in a significant reduction in G3 MB cell viability and proliferation. Together, these results highlight a critical role for the LIN28B–let‐7–PBK pathway in G3 MB and provide preliminary preclinical results for drugs targeting this pathway.

## Graphical abstract

Overexpression of *LIN28B* in Group 3 medulloblastoma cells promotes the upregulation of *PBK* and other oncogenes via inhibition of the microRNA let‐7. This results in increased proliferation of tumor cells and worse survival. Inhibitors of LIN28, such as compound 1632, or PBK, such as HI‐TOPK‐032, can lead to a reduction in tumor growth and cell proliferation.

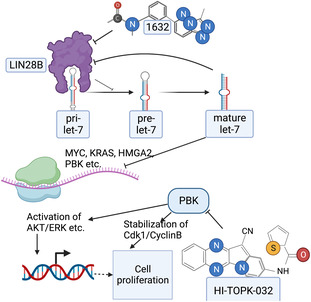

**Abbreviations**
AT/RT: atypical/teratoid rhabdoid tumor
BBB: blood–brain barrier
BrdU: Bromodeoxy uridine
CNS: central nervous system
DMSO: dimethyl sulfoxide
ETMR: embryonal tumor with multilayered rosettes
EGF: epidermal growth factor
FBS: fetal bovine serum
FGF: fibroblast growth factor
G3MB: Group 3 medulloblastoma
IC50: half maximal inhibitory concentration
HMGA2: high mobility group AT‐hook 2
HH3: histone H3
IGF: insulin‐like growth factor
LV105: lentiviral control
LVLIN28B: lentiviral LIN28B
let‐7: lethal‐7
LIN28A: LIN 28 homolog A
LIN28B: LIN 28 homolog B
MB: medulloblastoma
miRNA: microRNA
MAPKK: mitogen‐activated protein kinase kinase
HI‐TOPK‐032: *N*‐(12‐Cyanoindolizino[2,3‐b]quinoxalin‐2‐yl)‐2‐thiophenecarboxamide
1632: *N*‐Methyl‐*N*‐[3‐(3‐methyl‐1,2,4‐triazolo[4,3‐b]pyridazin‐6‐yl)phenyl]acetamide
PBK: PDZ‐binding kinase
pHH3: phospho‐histone H3
pRB: phospho‐Rb
PDGF‐AA: platelet‐derived growth factor AA
PDGF‐BB: platelet‐derived growth factor BB
qPCR: real‐time quantitative polymerase chain reaction
Rb: retinoblastoma
RNA: ribonucleic acid
RNA‐Seq: RNA sequencing
shctl: short hairpin control
shPBK: short hairpin PBK
shLIN28B: short hairpin RNA LIN28B
STR: short tandem repeat
RNU44: small nucleolar RNA 44
SHH: Sonic Hedgehog
TBP: TATA‐binding protein
WNT: wingless

## 1 Introduction

Medulloblastomas (MB) are the most common malignant pediatric tumor of the central nervous system. These tumors arise in the cerebellum, a brain region that continues to develop postnatally until approximately 2 years of age; this development is marked by rapid expansion of progenitor cell populations. Medulloblastoma was classified into four major molecular subgroups in 2011: Wingless (WNT), Sonic Hedgehog (SHH), Group 3, and Group 4. These subgroups differ in molecular pathogenesis, clinical associations, and prognosis [1]. Patients with Group 3 medulloblastoma (MB), especially those who have tumors that harbor *MYC* amplification or present with metastasis (along with a subset of SHH tumors with *TP53* mutation), have the worst survival outcome, while those with WNT subgroup tumors have the best survival [2]. Treatment for noninfant patients with MB includes maximal safe surgical resection followed by craniospinal irradiation and multiagent chemotherapy. Unfortunately, many patients with Group 3 MB have metastatic disease refractory to these treatments or develop resistance to treatment; therefore, more targeted therapies are clearly necessary for this group of patients. Although several molecular drivers of Group 3 MB have been identified, including *MYC*, *OTX2*, and *SMARCA4* [3], targeted therapies for Group 3 MB have not progressed to the clinic.

In 2014, Hovestadt et al. [4] demonstrated that the RNA‐binding protein *LIN28B* is overexpressed in Group 3 MB due to promoter hypomethylation and that this overexpression is associated with worse survival. More recently, Maklad et al. also demonstrated [5] that LIN28 is an important factor for medulloblastoma prognosis. However, the functional role of LIN28B has not been thoroughly investigated in Group 3 MB. *LIN28B* and its paralog *LIN28A* are well‐known cancer stem cell markers and have been demonstrated to drive growth and metastasis in several adult cancers, including breast, ovarian, esophageal, and pancreatic, as well as in pediatric neural tumors such as embryonal tumor with multilayered rosettes (ETMR), atypical teratoid/rhabdoid tumor (AT/RT), and neuroblastoma [6, 7, 8, 9, 10, 11, 12].

LIN28B is an inhibitor of maturation of the let‐7 miRNAs, which have been well characterized as a family of tumor suppressor miRNAs. As such, overexpression of *LIN28B* frequently leads to repression of let‐7 and subsequent de‐repression of let‐7 targets [13]. LIN28B and its downstream target PDZ‐binding kinase (*PBK*, also known as TOPK) are oncogenic in neuroblastoma and promote growth and self‐renewal via the LIN28B–let‐7–PBK axis [7]. PBK, a serine/threonine kinase belonging to the mitogen‐activated protein kinase kinase (MAPKK) family, is often overexpressed in cancers and is associated with poor prognosis [14]. More recently, *PBK* was identified as a hub gene in medulloblastoma by Deng et al. [15].

We hypothesized that the LIN28B–let‐7–PBK axis is an important driver of Group 3 MB progression and may be a novel therapeutic target in MB. To test this hypothesis, we genetically altered *LIN28B* expression in Group 3 MB cells and compared tumor cell viability and growth *in vitro* and *in vivo* and we determined the impact on downstream regulation of let‐7 and *PBK*. Finally, we investigated pharmacologic inhibition of LIN28B and PBK to explore the potential of targeting this pathway in the clinical setting. We report that *LIN28B* knockdown in a variety of Group 3 cell lines reduced cell proliferation by regulating let‐7 and *PBK*. We also demonstrate that the drugs 1632 and HI‐TOPK‐032 are effective in targeting LIN28B and PBK with evidence of *in vivo* efficacy for 1632.

## 2 Materials and methods

### 2.1 Cell lines

D341 (CVCL_0018), D556 (CVCL_1165), and CHLA01 (CVCL_B044) medulloblastoma cell lines were obtained from ATCC (USA). The BT52 and BT52CTC patient‐derived cells were provided by the Ian's Friend's Foundation Brain Tumor Biorepository at Children's Healthcare of Atlanta, Inc. The D425 (CVCL_1275) cell line was generously gifted by Dr. Eric Raabe, and the ONS‐76 (CVCL_1624) cell line was a gift from Dr. Charles Eberhart at Johns Hopkins University. The HDMB03 (CVCL_S506) cell line was generously gifted by Dr. Till Milde at the German Cancer Research Center (DKZF), Heidelberg. Mouse cerebellar astrocytes C8‐D1A type 1 clone (CVCL_6379) were purchased from ATCC. Lysates from the AT/RT cell line CHOA‐06 (CVCL_AQ42) were gifted by Dr. Andrew Hong. All cell lines were validated by short tandem repeat (STR) testing and routinely checked for mycoplasma. The patient‐derived cell lines were grown according to previously established protocols. Media for the cell lines were as follows: Eagle's Minimal Essential Medium (EMEM; Gibco, ThermoFisher Scientific, Waltham, MA, USA) supplemented with 20% fetal bovine serum (FBS) for D341, Dulbecco's Modified Eagle's Medium (DMEM; Gibco) supplemented with 10% FBS for D425, DMEM‐F12 supplemented with 20 ng·mL^−1^ EGF and 20 ng·mL^−1^ FGF for CHLA01, DMEM‐F12 supplemented with 10% FBS for ONS‐76, and Roswell Park Memorial Institute (RPMI) 1640 (Gibco) for HDMB03. Murine astrocytes were grown in DMEM supplemented with 10% FBS. For BT52 and BT52CTC, the cells were grown in a Neurobasal/DMEM‐F12‐based base medium supplemented with a growth factor mix. The base medium is a 50 : 50 mix of Neurobasal A (Lonza, Basel, Switzerland) DMEM‐F12 (Life Technologies, ThermoFisher Scientific, Waltham, MA, USA) and 1× each of sodium pyruvate, Minimum Essential Media (MEM) nonessential amino acids, HEPES (*N*‐2‐hydroxyethylpiperazine‐*N*‐2‐ethane sulfonic acid), and glutamax. The 10× growth factor mix is made of base medium (71% w/v) with B27 without vitamin A (1×), IGF (100 ng·mL^−1^), EGF (50 ng·mL^−1^), FGFb (40 ng·mL^−1^), PDGF‐AA (20 ng·mL^−1^), PDGF‐BB (20 ng·mL^−1^), and 0.2% heparin (2 μg·mL^−1^).

### 2.2 RNA‐Seq expression analysis

RNA sequencing was carried out on human medulloblastoma samples via the Medulloblastoma Advanced Genomics International Consortium (MAGIC) as described previously [16]. Briefly, fresh tumor samples were collected after receiving written informed consent per the ethical regulations of the contributing institutions and stored at −80 °C until being processed for nucleic acid purification. The cohort consisted of 66 WNT, 279 SHH, 192 Group 3, and 334 Group 4 tumors. Nine control samples were obtained from normal adult (*n* = 5) and fetal (*n* = 4) cerebellar tissue. PolyA‐enriched, 100‐bp paired‐end sequencing reads were generated on Illumina HiSeq2000 or 2500 and mapped by STAR version 2.5.1b to the human reference genome ‘hs37d5’ [17]. Read counts were normalized using variance‐stabilizing transformation with the DESeq2 R Bioconductor package [18]. Expression of *LIN28B* was compared across all samples between groups and statistical significance was assessed using the Wilcoxon rank‐sum test. The MAGIC cohort data [16] were also used to analyze correlation between *LIN28B* expression and *TP53* mutation status. In brief, we compared *LIN28B* expression between SHH tumors with *TP53* mutations (DEL, INS, and SNP) or WT.

### 2.3 Plasmids, lentiviral transduction, and selection

Plasmids targeting *LIN28B* (shLIN28B‐1 TRCN0000122191; shLIN28B‐5 TRCN0000219859), *PBK* (shPBK‐1 TRCN0000001806; shPBK‐2 TRCN0000001805), and corresponding control (shctl shc002; pLKO.1 lentiviral backbone empty vector) were purchased from Millipore Sigma (Merck KGaA, Dermstadt, Germany). LIN28B overexpression plasmid (LVLIN28B; Y3355) and control (LV105; EX‐Y3355‐Lv105) were purchased from Genecopoeia (Rockville, MD, USA). Plasmids were amplified in E.coli Stbl3 cells (Invitrogen, Carlsbad, CA, USA) following the manufacturer's protocols. After isolating plasmids using maxiprep technique, plasmids were packaged in lentivirus in HEK293T cells with pMD2.G, psPAX2 (Addgene, Watertown, MA, USA) following previously described protocols [4, 7]. Briefly, 3 μg of each plasmid is added to 80% confluent HEK293T plates along with Fugene transfection reagent (Promega, Madison, WI, USA). Supernatant containing virus is collected at 48 and 72 h and then stored at −80 °C until viral transfection. On the day of infection, Group 3 MB cells are plated at low density on 6‐well plates and 4 mL aliquot of virus is added to media. Antibiotic selection is started after 72 h. For selection using puromycin, we used the following concentrations: 2 μg·mL^−1^ for BT52, BT52CTC, and HDMB03, 3 μg·mL^−1^ for D425, and 5 μg·mL^−1^ for D341.

### 2.4 Tumor sphere formation assay

D556 cells were detached and then plated at low density (5000 cells per well) in 96‐well plates in tumor sphere media (DMEM/F12 supplemented with 20 ng·mL^−1^ EGF and 20 ng·mL^−1^ bFGF) and left undisturbed for 10 days before spheres were imaged using Lionheart FX microscope and then counted using imagej software (NIH, Bethesda, MD, USA).

### 2.5 miRNA mimic transfection

Mature let‐7i‐5p mimic (C‐300584‐05‐0002) and scrambled negative control (CN‐001000‐01‐05) were purchased from Dharmacon (GE healthcare, Lafayette, CO, USA) and transfected following the manufacturer's protocols using Lipofectamine 2000 (Invitrogen). Cells were incubated in media containing 10 nm mimic or negative control for 48–72 h before collecting cell pellets for RNA and protein isolation.

### 2.6 Real‐time PCR

RNA was isolated using the Zymo RNA isolation kit following the manufacturer's recommendations. cDNA was synthesized using the High Capacity cDNA reverse transcription kit (Thermo Fisher, Waltham, MA, USA). For mRNA qPCR, the primers were as follows: hLIN28B‐FW: CCTTGGATATTCCAGTCGATGTAT; hLIN28B‐RV: TGACTCAAGGCCTTTGGAAG. hTBP‐FW: GCTGAGAAGAGTGTGCTGGA; hTBP‐RV: TAAGGTGGCAGGCTGTTGTT; hACTIN‐FW: GGGCATGGGTCAGAAGGATT; hACTIN‐RV: TCGATGGGGTACTTCAGGGT. qPCR was carried out following standard protocols using SYBR Green (Bio‐Rad, Hercules, CA, USA) reagent.

For miRNA qPCR analysis, reverse transcription and real‐time PCR were carried out following the method described in Niu et al. [19]. Small nucleolar RNA RNU44 was used as housekeeping gene for miRNA qPCR and TATA‐binding protein (TBP) for mRNA qPCR. All primers for reverse transcription and qPCR are listed in Table S1.

### 2.7 Immunoblotting

Cells collected for protein isolation were immediately placed in RIPA lysis buffer with protease and phosphatase inhibitors and PMSF. Lysates are collected after vortexing and brief sonication and a 30‐min high‐speed spin at 4 degrees. Once collected, lysates are stored until ready for loading into gels or protein quantification. Gels are prepared and run following standard protocols. Primary antibodies against LIN28B (#11965), PBK (#4942), Phospho‐PBK (#4941), LIN28A (#8641), Ku80 (#2753), PARP (#9542), phospho‐Rb (pRb) (Ser780) (#9307), Rb (D20) (#9313), histone H3 (D1H2) (#4499), phospho‐histone H3 (pHH3) (Ser10) (D7N8E) (#53348), HMGA2 (#8179), and Kras (#53270) were purchased from Cell Signaling Technology (Danvers, MA, USA). Antibodies against ß‐tubulin (sc‐53 140) and c‐Myc (9E10) (sc40) were obtained from Santa Cruz Biotechnology (Dallas, TX, USA). Densitometry analysis was performed using imagej software. For each protein of interest, area under the curve from intensity was calculated and then normalized to loading control (Tubulin or Ku80). Phospho‐proteins and cleaved PARP specifically were normalized to corresponding total proteins as well.

### 2.8 Drugs

We purchased LIN28B 1632 (Cayman Chemical, Ann Arbor, MI, USA), and HI‐TOPK‐032 (Tocris Bioscience, Bristol, UK) from suppliers and dissolved in Dimethyl sulfoxide (DMSO) before storing aliquots at −80 °C. For drug dose studies, the amount of DMSO required for the maximum drug concentration is used as a control (DMSO only).

### 2.9 Animal studies

All animal studies were carried out under the guidance of the Emory Institutional Animal Care and Use Committee. NOD SCID gamma (NSG) mice were obtained from The Jackson Laboratory (005557) (Bar Harbor, ME, USA). All mice are housed in sterile micro‐isolators, and sentinels are routinely screened for specific pathogens. In addition to veterinary care, facility personnel also perform routine maintenance of mouse colonies. Mice are kept in sterile cages with no more than 5 mice per cage and are only handled in sterile hoods except at the time of euthanasia. All personnel handling mice wore personal protective equipment in the form of gloves and gown and were only allowed in the animal facility after completing required training and addition to IACUC protocol. For survival studies, approximately 20 000 cells with either *LIN28B* knockdown or control transfection (shctl) were injected in the posterior fossa of NSG pups between p2 and p7 (postnatal day) and mice were followed until they developed signs of tumor burden or intracranial pressure (hunching, cachexia, ataxia, seizures, or head swelling). Mice were then humanely euthanized and the brains fixed for immunohistochemistry in formalin or flash frozen for protein isolation. For flank tumor injections, 1 × 10^6^ HDMB03 cells or 1.5 × 10^5^ D341 cells mixed 5 : 1 with Matrigel (Corning, Corning, NY, USA) were injected into each rear flank of 4–6‐week‐old NSG mice. Equal numbers of male and female mice were injected. Each flank was injected with 40 mg·kg^−1^ of 1632 dissolved in ethanol or vehicle control (ethanol alone) on Monday, Wednesday, and Friday for five or six total doses (over 10–12 days) starting 2 weeks after cell injection. Mice were monitored for signs of tumor burden such as skin discoloration, ulcerations, gait changes, or behavior changes and were sacrificed after 12 days when tumors reached a predetermined maximum dimension of 2 cm in any direction. For HDMB03 injections, tumors were measured once at the beginning and again at the end of 12 days. For D341, tumors were measured twice a week. Mice tolerated intratumoral injection of 1632 without any signs of toxicity or loss of body weight (weighed 2× per week). All animals were euthanized following Emory IACUC's approved protocol before collecting flanks for imaging and freezing tissue for immunoblots.

### 2.10 Immunohistochemistry

NSG mice orthotopically injected with D425 or D341 cells transfected with either control plasmid (shctl) or LIN28B shRNA (shLIN28B) were sacrificed 4 weeks after injection. Following euthanasia, whole brains were placed in formalin. Tumor sections from de‐identified patient samples stored in Children's Healthcare of Atlanta biorepository with histopathologically confirmed MYC‐amplified Group 3 MB were obtained at the time of initial resection after obtaining informed consent per Children's Healthcare of Atlanta (CHOA) IRB‐approved protocol (IRB00034535), and slides cut from paraffin blocks before immunostaining.

For *ex vivo* drug treatment, whole brains (unfixed) from mice with D341 orthotopic tumors were embedded in 4.5% (w/v) low melting agarose and then sliced into 300 μm sections using a vibratome. Sections were then cultured in DMEM/F12 media for 1 day before treatment with drug or vehicle control. After 48 h, slices were fixed in 4% paraformaldehyde and then embedded in paraffin.

Formalin‐fixed, paraffin‐embedded brain tissue sections from each group were cut to a 5‐μm thickness and air‐dried. Staining was performed using Ventana DISCOVERY Ultra automated immunohistochemistry device (Ventana Medical Systems, Tucson, AZ, USA). Slides were deparaffinized with EZ‐Prep (# 05279771001, Ventana) and then were antigen retrieved for 40 min with CC1 reagent (#950‐500, Ventana). Rabbit anti‐LIN28B (CST #4196), anti‐Ki67 (Abcam ab16667, Cambridge, UK), anti‐cleaved caspase‐3 (CST #9661), and anti‐c‐MYC (Abcam ab32072) antibodies diluted at 1 : 100 and anti‐PBK (CST #4942) diluted at 1 : 50 were applied and incubated for 40 min. DISCOVERY OmniMap anti‐Rb HRP was applied and incubated for 12 min, and the detection was completed in combination with DISCOVERY ChromoMap DAB kit, as per the manufacturer's recommendations. Slides were counterstained with hematoxylin for 12 min. Slides were then dehydrated, cover‐slipped, and evaluated by light microscopy. The fraction of positive cells in each figure was counted in representative fields using imagej software (# of stained cells × 100)/total # of cells in tumor area).

### 2.11 Cell viability assay

Cells were plated at low density (1000–2000/well) in 96‐well optical plates. For drug treatment studies, cells were treated with either drug or vehicle control. After 96 h, CellTiter‐Glo reagent (Promega) is added at equal volume. Optical density is measured with a plate reader and luminescence plotted using Graphpad Prism (Graphpad Software, San Diego, CA, USA) software.

### 2.12 Immunofluorescence

Immunofluorescence analysis of MB cells was performed as described previously [20]. Briefly, cells were pulsed with BrdU (Millipore Sigma) for 1 h before harvesting and fixation. Cells were then cytospun onto a glass slide. Cells were permeabilized, and the DNA was hydrolyzed with 2 N HCl and incubated with primary anti‐BrdU antibody (CST #5292) overnight, before incubating with fluorescently tagged secondary antibody (Invitrogen‐A11005). For each slide, cells were imaged in at least three fields (up to 10 fields) and BrdU‐positive cells were counted.

### 2.13 Flow cytometry

Cells plated at equal density were either treated with drug or vehicle and allowed to grow for 72 h before pulsing with BrdU for 1 h for BrdU incorporation assay or harvested in the case of Annexin V assay. Cells were then washed and processed following protocols described in the BD APC BrdU flow kit (BD Pharmingen, San Diego, CA, USA) for BrdU assay. For the Annexin V assay, cells were washed in PBS before counting and staining with FITC Annexin V and propidium iodide (PI) and then analyzed by flow cytometry. All flow cytometry data were analyzed by FlowJo software (Becton Dickinson and Company, Franklin Lakes, NJ, USA).

### 2.14 Statistical methods

All experiments were done with at least three biological replicates. Statistical analysis was done using graphpad prism 9. Statistical significance was calculated using Wilcoxon rank‐sum test, Student's *t*‐test, and log‐rank test.

### 2.15 Ethical statement

Tumor sections from de‐identified patient samples stored in Children's Healthcare of Atlanta biorepository with histopathologically confirmed MYC‐amplified Group 3 MB were obtained at the time of initial resection after obtaining informed consent per CHOA IRB‐approved protocol (IRB00034535). The study conforms to standards set by the Declaration of Helsinki. The experiments were undertaken with the understanding and written consent of each subject. All animal studies described in this manuscript were carried out under the guidance of the Emory Division of Animal Resources and were approved by Emory IACUC within protocol 201700740.

## 3 Results

### 3.1 *LIN28B* and not *LIN28A* is expressed in Group 3 MB tumors and cell line models

**Fig. 1:**
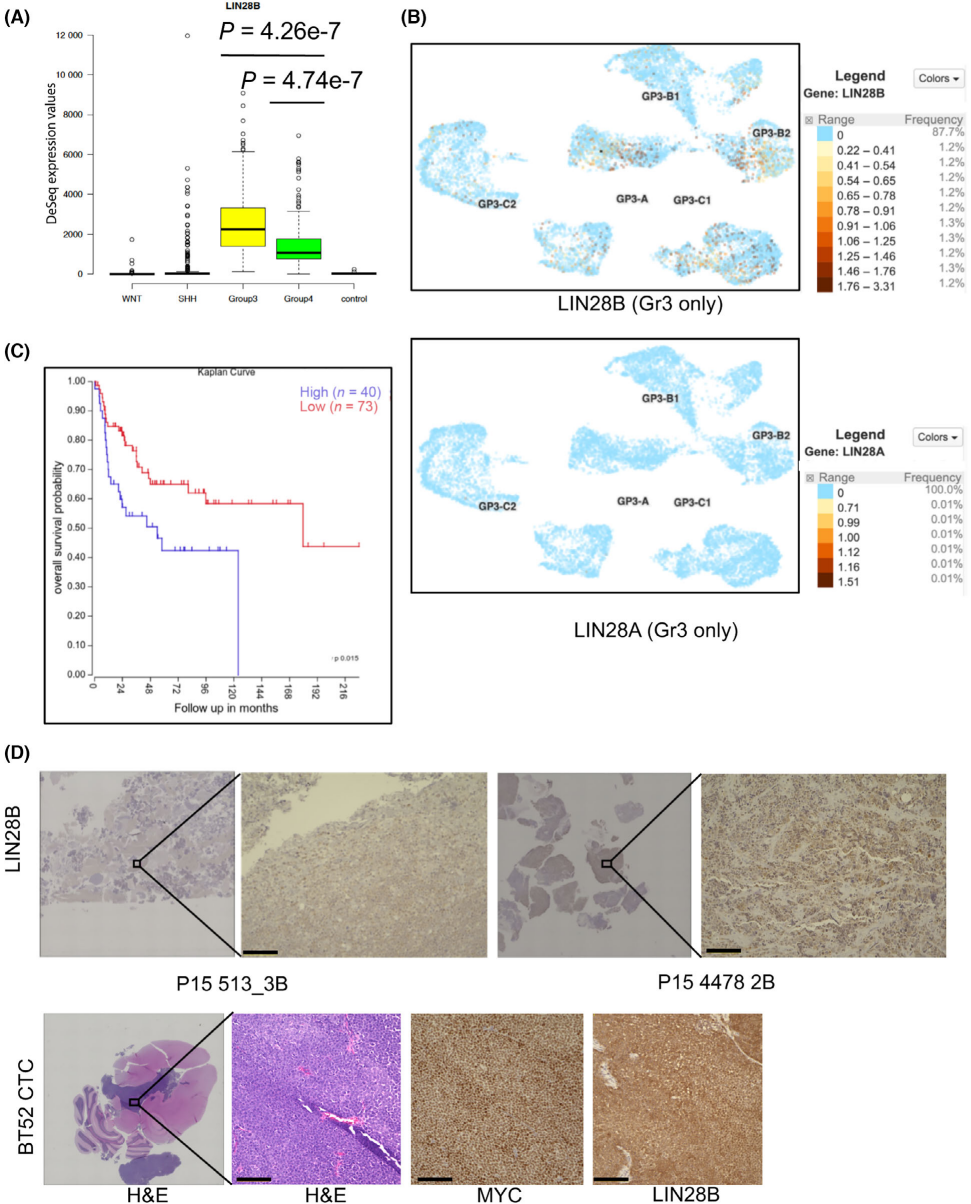
LIN28B is the predominant form of LIN28 in Group 3 medulloblastoma with prognostic significance. (A) Normalized read counts of RNA‐Seq analysis (using DeSeq2 package) from patients with medulloblastoma demonstrate that *LIN28B* is significantly overexpressed in Group 3 and (to a lesser extent) Group 4 MB patients compared with controls (cerebellar RNA from epilepsy patients; Wilcoxon rank‐sum test *P*‐value of Group 3 vs control and Group 4 vs control are noted; box limits demonstrate 25^th^ and 75^th^ percentile. Lower and upper whiskers: extend 1.5 times the interquartile range from the 25^th^ and 75^th^ percentiles. Individual points: Outliers). (B) UMAP plot of single‐cell transcriptome analysis from 7 GP3 MB patients with relative expression of *LIN28B* (top) or *LIN28A* (bottom) (GP3‐A [mitotic], B1, B2 [progenitor], C1 and C2 [differentiated] refer to subclusters defined by Riemondy et al. [22]). (C) Increased expression of *LIN28B* is associated with worse survival in Group 3 MB patients (chi‐squared test *P*‐value 0.015; Data downloaded from R2 portal; low vs high expression defined by built‐in software KaplanScan with cutoff at 96.8) [42]. (D) Tumor sections from two patients with MYC amplified Group 3 MB demonstrating representative areas with LIN28B stain (scale bar = 100 μm) (top row). H&E (10× and 20×), MYC, and LIN28B stain of PDOX brain slice derived from BT52CTC patient cells (scale bar = 100 μm) (bottom).

**Fig. 2:**
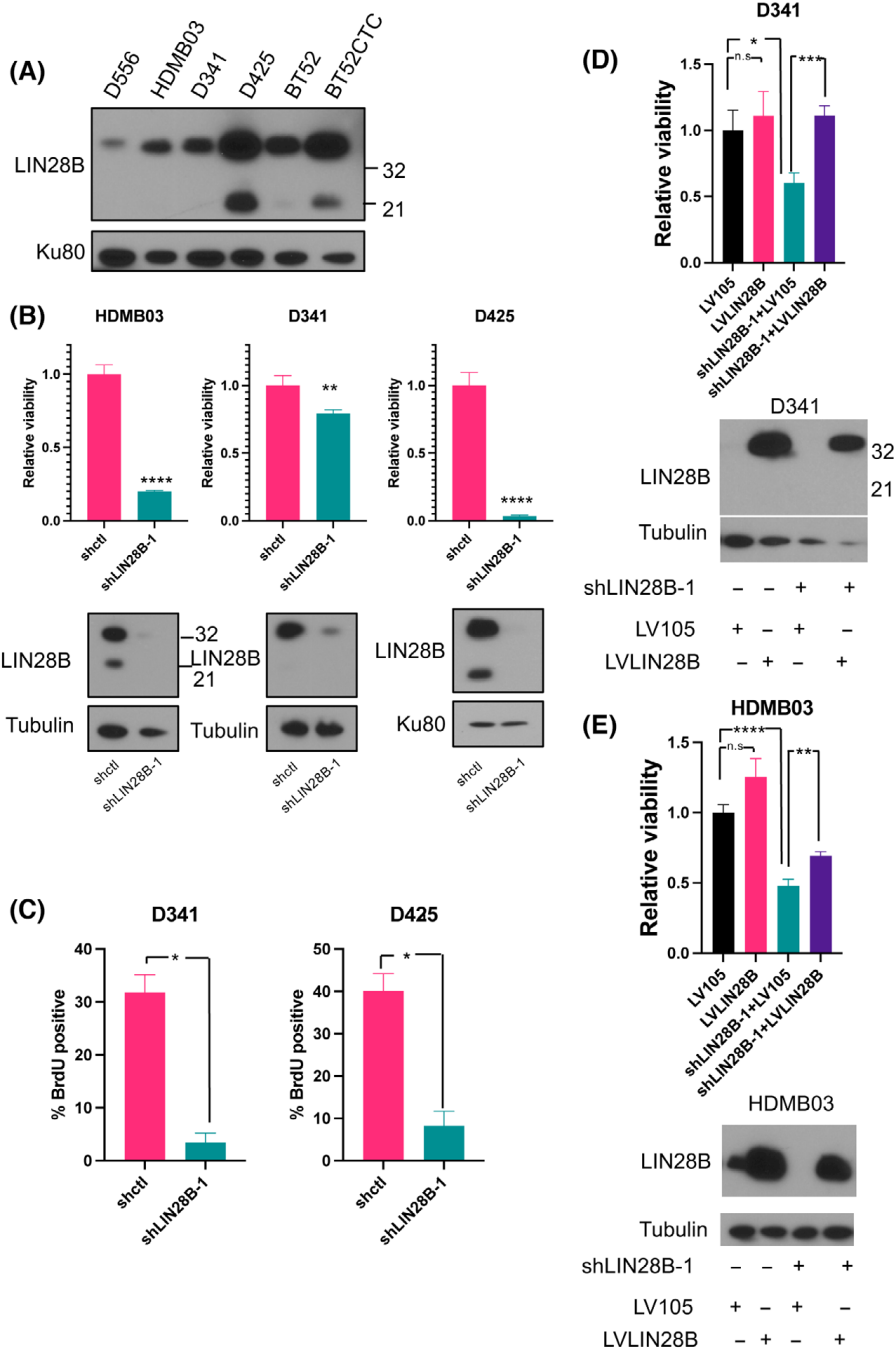
LIN28B promotes Group 3 medulloblastoma viability and proliferation. (A) Relative levels of LIN28B protein in Group 3 MB cell lines D556, HDMB03, D341, and D425 and patient‐derived cells BT52 and BT52CTC. (B) CellTiter‐Glo assay demonstrates a significant reduction in viability in shLIN28B cells compared with shctl cells. LIN28B levels in HDMB03, D341, and D425 cells treated with either control shRNA (shctl) or *LIN28B* shRNA (shLIN28B‐1) demonstrate reduction in LIN28B following shRNA transfection (see Fig. S2 for experiments using a 2^nd^ shRNA (shLIN28B‐5)). (C) BrdU incorporation analysis using immunofluorescence in Group 3 MB cell lines demonstrates a significant decrease in BrdU‐positive cells following *LIN28B* knockdown (*n* = 3 for each group). (D) In D341 and (E), HDMB03 shLIN28B‐1 knockdown cells LIN28B rescue leads to restoration of cell viability. Western blot of corresponding cell lysates demonstrating rescue of LIN28B protein in knockdown cells (LV105 overexpression plasmid control; LVLIN28B *LIN28B* expression plasmid) is shown below (unpaired *t*‐test *P*‐value: *P < 0.05; **P < 0.01; ***P < 0.001; ****P < 0.0001; *n* = 3 for each group). Error bars represent SEM.

LIN28 has been shown to be an important regulator of pluripotency and metastasis in several cancer models [6]. LIN28 has two paralogs in humans. While both *LIN28A* and *LIN28B* can act as oncogenes, we sought to investigate which of these were predominant in Group 3 MB. Bulk RNA‐Seq data and microarray from patients in multiple data sets (Fig. 1A, and [21]) and single‐cell RNA‐Seq data obtained from patients [22] demonstrated that *LIN28B*, but not *LIN28A*, is significantly overexpressed in Group 3 tumors compared with normal cerebellum (Fig. 1A,B and Fig. S1a,b). Of note, LIN28B has two isoforms (approximately 21 and 32 kDa), and while all Group 3 cells express the larger isoform, some express the smaller isoform in much lower amounts. Overexpression of *LIN28B* is also associated with poor prognosis in medulloblastoma (Fig. 1C; and [4, 5]); however, within the SHH subgroup, there was no difference in *LIN28B* expression between p53 wild‐type and p53 mutant tumors, which have a worse prognosis, suggesting differential functions for LIN28B in the SHH subgroup (Fig. S1c). We analyzed tumor sections from patients with MYC amplified Group 3 MB as well as a patient‐derived orthotopic xenograft (PDOX) utilizing the BT52CTC patient cells and confirmed expression of LIN28B in these tumors (Fig. 1D and Fig. S1d). While we do not see a direct correlation between MYC and LIN28B expression at the cellular level in these tumors, both of the patients had MYC amplification confirmed by pathology and they both expressed LIN28B. Finally, we explored both patient‐derived MB cell lines and primary cells and found that LIN28B is present at robust levels in most Group 3 MB cells, while LIN28A is not (Fig. 2A and Fig. S1e). These findings suggest that in Group 3 MB, LIN28B, and not LIN28A, is the predominant form of functional LIN28.

### 3.2 LIN28B promotes Group 3 MB proliferation and tumor growth

Since high *LIN28B* expression has been postulated to be a dependency in Group 3 MB [23, 24, 25] and is associated with poor prognosis in these patients (Fig. 1C and [4]), we investigated whether reduction in LIN28B levels could inhibit tumor growth. To this end, we used shRNAs targeting *LIN28B* to decrease LIN28B levels in Group 3 MB cells (Fig. 2B, and Fig. S2a–c) and found that this led to a significant decrease in cell viability by CellTiter‐Glo (Fig. 2B and Fig. S2a). Both LIN28A and LIN28B have previously been demonstrated to regulate cell cycle entry [26, 27]. To investigate whether LIN28B promotes proliferation of Group 3 MB cells by regulating cell cycle progression, we performed immunofluorescence to detect BrdU incorporation following *LIN28B* knockdown. Our results demonstrate a significant reduction in BrdU incorporation, indicating a decrease in S‐phase entry following *LIN28B* knockdown (Fig. 2c and Fig. S3). We obtained similar results with a different shRNA (shLIN28B‐5; Figs S2d and S3), as well as by flow cytometry (data not shown). Conversely, when we rescued LIN28B levels in the knockdown Group 3 MB cells with a *LIN28B* overexpression plasmid, we saw increased cell viability (Fig. 2D). Increased cell viability was also observed with forced overexpression of *LIN28B* in the wild‐type D556 Group 3 MB cell line (Fig. S2e), which expresses very low levels of LIN28B at baseline (Fig. 2A). *LIN28B*‐overexpressing cells formed significantly more tumor spheres than control lentivirus (LV105) expressing D556 cells (Fig. S2e). Taken together, these results indicate that LIN28B plays an important role in Group 3 MB viability and proliferation.

### 3.3 LIN28B regulates the let7–PBK pathway in Group 3 MB cells through inhibition of let‐7

**Fig. 3:**
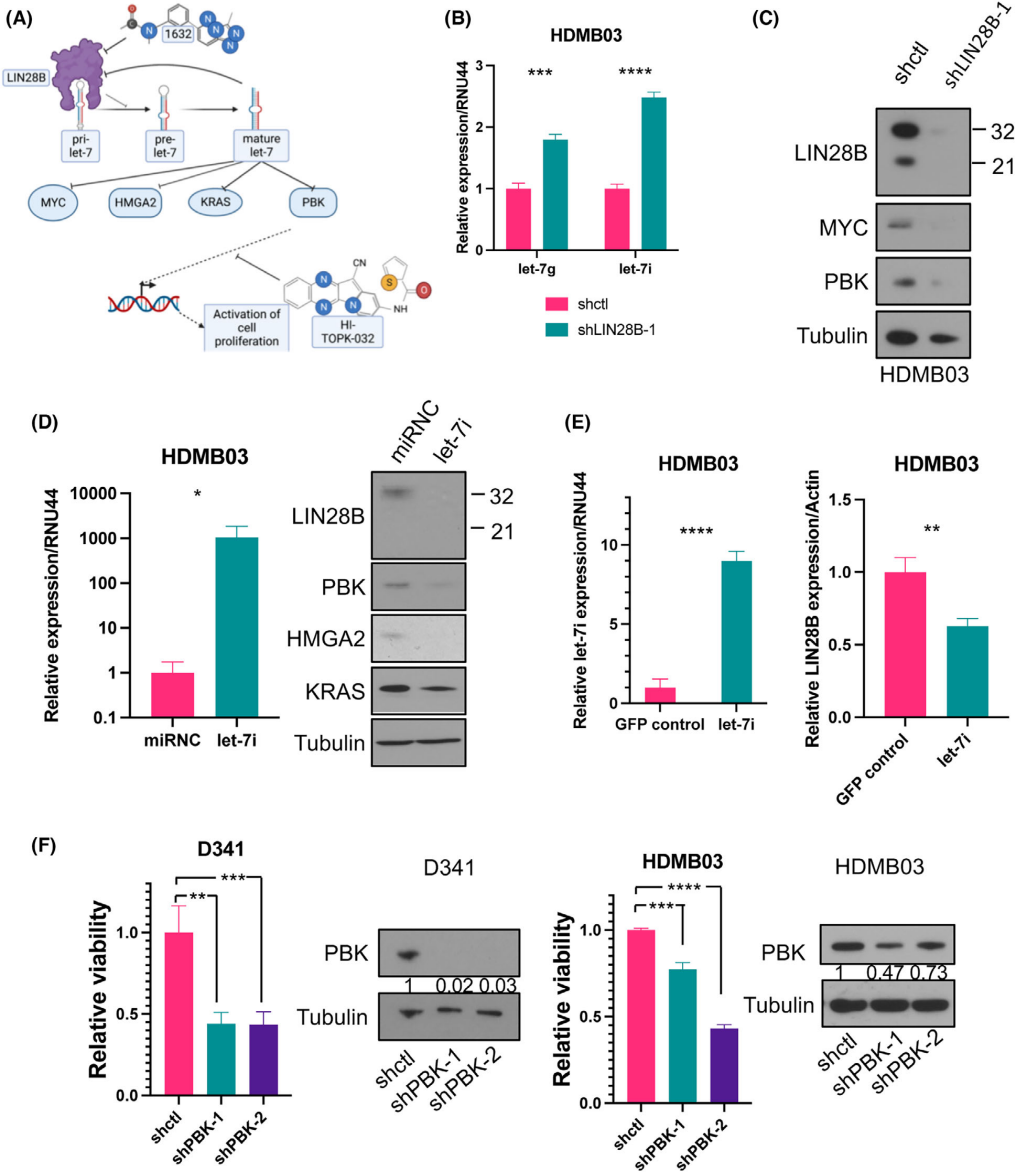
LIN28B regulates let‐7 and PBK. (A) Schematic of the LIN28B‐let‐7 pathway demonstrating LIN28B inhibits let‐7 maturation. In turn, let‐7 inhibits PBK, HMGA2, KRAS, MYC, and LIN28B. Compound 1632 and HI‐TOPK‐032 are also shown which inhibit the LIN28B‐let‐7 interaction and PBK activity respectively. (B) *LIN28B* knockdown in HDMB03 cells leads to increased *let‐7i* and *let‐7g* expression in the shLIN28B treated cells compared with controls (RNU44 used as internal reference gene). (C) Western blot showing decreased levels of PBK and MYC protein in shLIN28B‐1‐treated cells compared with cells treated with control shRNA (shctl). (D) Transient overexpression of *let‐7i* in HDMB03 cells leads to decreased levels of LIN28B, PBK, HMGA2, and KRAS as demonstrated by western blot. (E) Stable transfection of *let‐7i* leads to decreased expression of *LIN28B*. (F) CellTiter‐Glo assay demonstrates a significant reduction in cell viability and western blot demonstrates reduction in PBK levels in D341 and HDMB03 cells following PBK knockdown (shPBK: shRNA targeting PBK, shctl: control shRNA; Numbers under blots represent densitometric analysis relative to loading control and normalized to control lane; Unpaired *t*‐test *P*‐value: *P < 0.05; **P < 0.01; ***P < 0.001; ****P < 0.0001; *n* = 3 for each group unless otherwise stated). Error bars represent SEM.

LIN28B promotes neuroblastoma growth and metastasis through its regulation of the let‐7–PBK axis [7]. LIN28B is known to inhibit the maturation of microRNAs (miRNAs) of the let‐7 family, which inhibit the translation of several oncogenes including *LIN28B* itself, *PBK*, *HMGA2*, and *KRAS* [6, 28] (Fig. 3A). Thus, decreased LIN28B is predicted to result in upregulation of let‐7 and downregulation of PBK, KRAS, and other let‐7 targets.

To investigate whether LIN28B regulates the let‐7–PBK pathway in Group 3 MB cells, we collected RNA and protein lysates from *LIN28B* knockdown cells and performed qPCR for *let‐7* and immunoblotting for let‐7 targets (e.g., PBK and MYC). As expected, qPCR analysis of *LIN28B* knockdown cells showed significant upregulation of *let‐7g* and *let‐7i* compared with controls (Fig. 3B and Fig. S4a). We found that knockdown cells had significantly lower levels of PBK and other targets compared with controls (Fig. 3C and Fig. S4b). Transient transfection of the let‐7i‐5p miRNA mimic (10 nm) in the Group 3 MB cell lines caused marked upregulation (~ 1000×) of let‐7i by qPCR (Fig. 3D and Fig. S4c), and consequently, significant downregulation of PBK, HMGA2, and KRAS, as well as LIN28B protein levels (Fig. 3D and Fig. S4d). Importantly, stable transfection of HDMB03 cells with a let‐7i hairpin also led to upregulated *let‐7i* expression that was more physiologic (~ 9×) and downregulation of LIN28B levels (Fig. 3E). Finally, we used shRNA to target *PBK* in Group 3 MB cell lines (Fig. 3F) and primary patient cells (Fig. S4e) and found that downregulation of PBK resulted in decreased viability similar to *LIN28B* knockdown. These results indicate that LIN28B promotes Group 3 MB viability and proliferation by regulating the let‐7–PBK axis.

### 3.4 Pharmacologic inhibition of LIN28B decreases Group 3 MB cell proliferation by inhibiting cell cycle entry

**Fig. 4:**
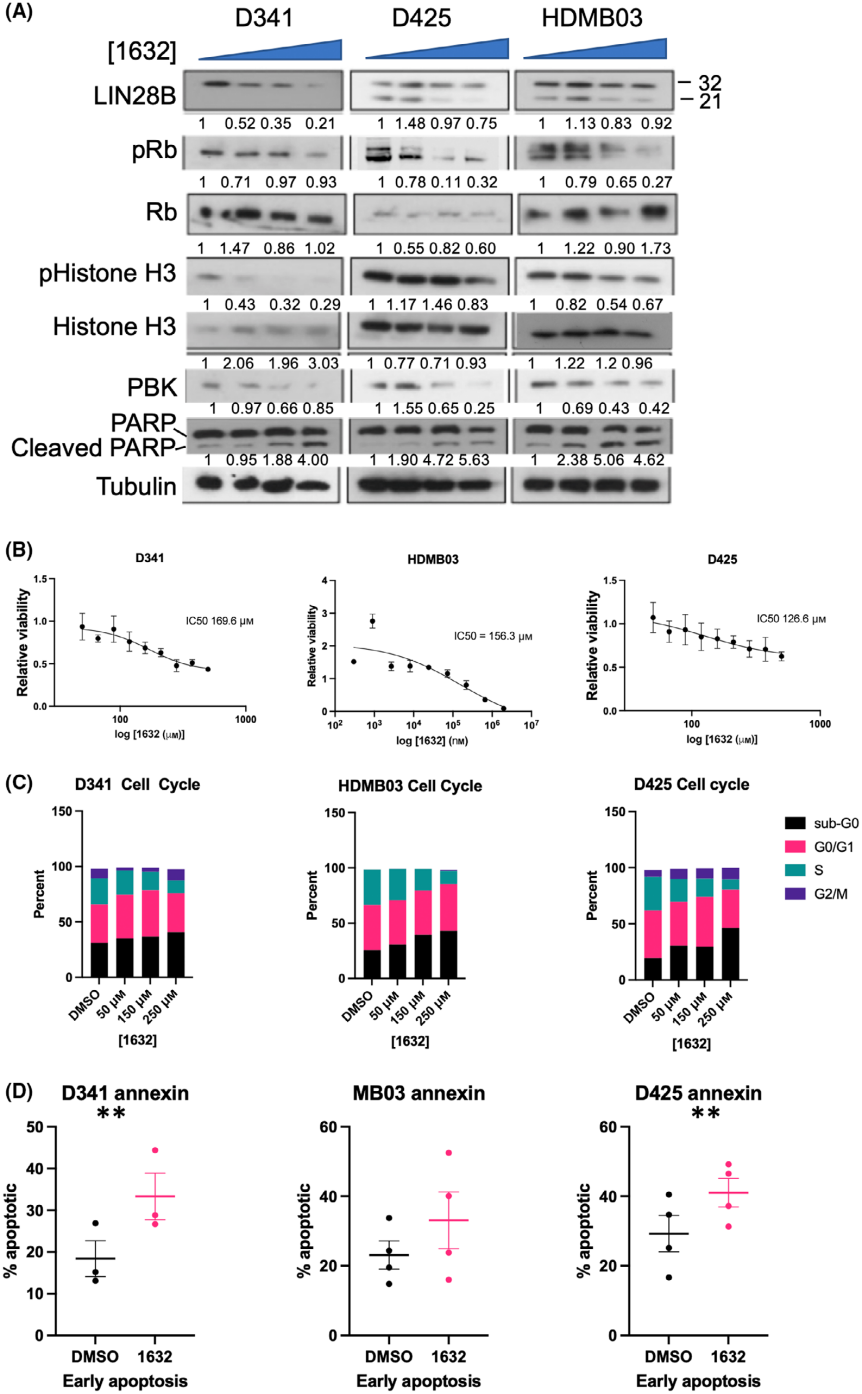
LIN28 inhibitor compound 1632 significantly decreases Group 3 medulloblastoma viability and proliferation. (A) Western blot showing decreased levels of LIN28B, PBK, phospho‐Rb, phospho‐histone H3 and increasing cleaved PARP expression following treatment with increasing doses of 1632 drug concentration (from left to right) DMSO, 50, 100, 250 μm (numbers under blot represent densitometric analysis relative to loading control and normalized to control lane). (B) Decrease in Group 3 MB cell viability with increasing concentration of 1632. IC50 for D341, D425, and HDMB03 is noted (only representative image shown, experiment replicated with at least three biological replicates; error bar represents SEM calculated from six technical replicates). (C) Cell cycle analysis with flow cytometry using BrdU incorporation 72 h following 1632 drug treatment demonstrates a dose‐dependent decrease in cell cycle entry (%S) and increase in Sub G0/G1 population in D341, D425, and HDMB03 cells (Only representative graph shown, see Table S2 for *P*‐value). (D) Annexin V stain for apoptosis demonstrates increased apoptosis following 250 μm 1632 treatment for 72 h in D341, D425 (Unpaired *t*‐test *P*‐value: **P < 0.01), and HDMB03 cells (n.s.; *n* = 3 for each group unless otherwise stated). Error bars represent SEM.

**Fig. 5:**
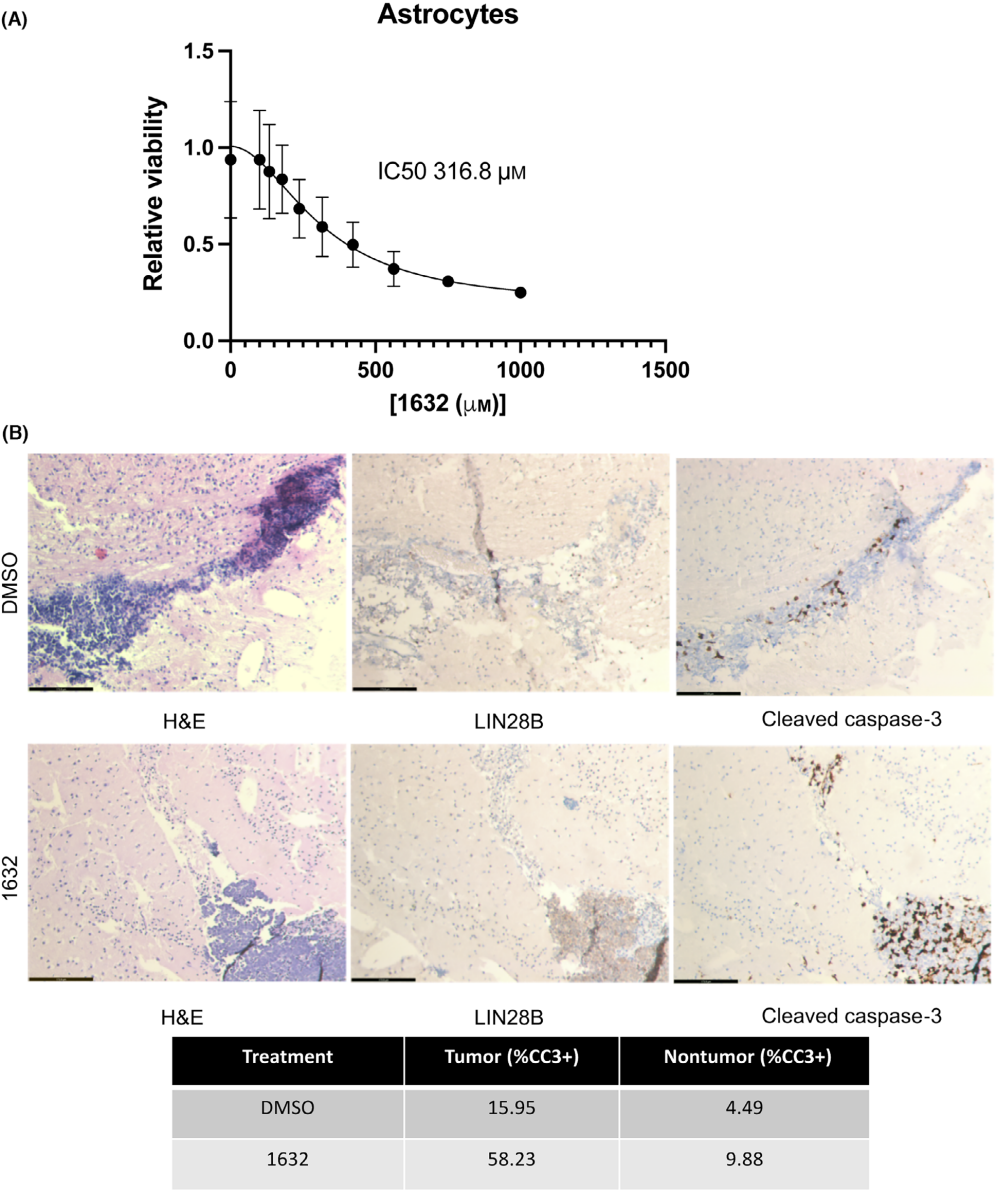
LIN28 inhibitor 1632 is not toxic to normal astrocytes and other nontumor cells. (A) Dose‐dependent reduction in mouse astrocyte viability following treatment with 1632. IC50 calculated from nonlinear fit (regression) analysis for this replicate is demonstrated (error bar represents SEM calculated from six technical replicates; additional experiments used to establish range reported in text, average calculated from three biological replicates). (B) H&E, LIN28B, and cleaved caspase‐3 staining of orthotopic brain slices derived from D341 cells treated with either DMSO or 250 μm 1632. Percentage of cleaved caspase‐3 (CC3)‐positive cells in the tumor and nontumor areas are shown (scale bar = 174 μm). Error bars represent SEM.

Recently, a pharmacologic inhibitor of LIN28B, ‘compound 1632’, was identified that demonstrated preclinical efficacy in a LIN28B‐positive Ewing sarcoma model [29] as well as other cancers [30, 31]. We postulated that this agent would demonstrate similar efficacy in Group 3 MB cells. Indeed, when we treated Group 3 MB cells with 1632 (50–250 μm), we observed a dose‐dependent decrease in LIN28B, PBK, phospho‐histone H3 (pHH3), and phospho‐Rb (pRb), and an increase in cleaved PARP (Fig. 4A). We also observed a dose‐dependent decrease in cell viability (Fig. 4B). We next investigated whether the effect of this drug on proliferation was primarily due to promoting apoptosis or cell cycle arrest. As in our *LIN28B* knockdown studies in the Group 3 MB cells, we detected a dose‐dependent decrease in Group 3 MB cells entering S‐phase and an increase in Sub‐G0/G1 population following drug treatment (Fig. 4C and Table S2). To demonstrate that the effect of 1632 is specific to LIN28B, we treated our *LIN28B* knockdown cells with 250 μm 1632 (Fig. S5) and found that cells with the most effective knockdown did not show any incremental reduction in viability with the addition of 1632, suggesting specificity rather than off‐target effects at these doses. To investigate whether 1632 leads to an increase in apoptosis, we performed annexin V/PI staining in Group 3 MB cells treated with 1632 and found only a slight increase after drug treatment, arguing against drug‐induced cell death being a major mechanism for 1632's effect on Group 3 MB cells (Fig. 4D). To investigate whether these doses will be tolerable to normal CNS cells, we treated murine cerebellar astrocytes with 1632 and found that the IC50 was significantly higher at 321.4 μm (range 275–371 μm) (Fig. 5A).We also treated D341 orthotopic tumor slices with 250 μm 1632 *ex vivo* and found that 1632 treatment increased the proportion of cleaved caspase‐3‐positive cells, and importantly, the increase in cleaved caspase‐3 staining was primarily in the tumor area while sparing the adjacent nontumor area (Fig. 5B). These results indicate that targeting LIN28B using compound 1632 causes decreased proliferation of Group 3 MB cells, which is primarily mediated by reduced cell cycle entry and that this treatment should be well tolerated in nontumor areas of the brain.

### 3.5 LIN28B promotes Group 3 MB tumor growth *in vivo*

**Fig. 6:**
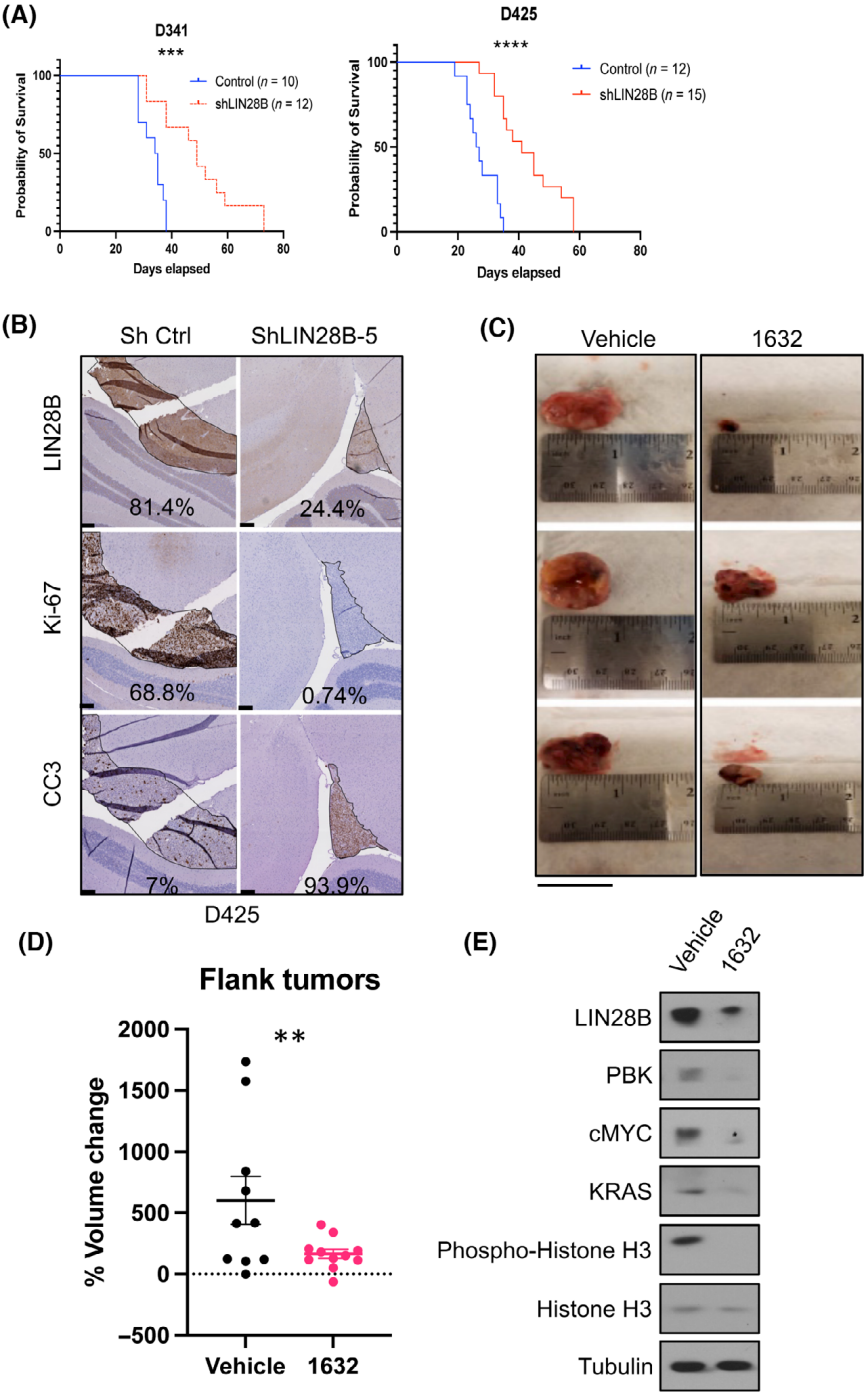
LIN28B inhibition prolongs survival and decreases tumor size *in vivo*. (A) Survival plots of mice orthotopically injected with D341 or D425 cells stably transfected with shctl or shLIN28B [log‐rank (Mantel‐Cox) test *P*‐value: ***P < 0.001; ****P < 0.0001]. (B) Immunohistochemistry demonstrating LIN28B, Ki‐67, and cleaved caspase‐3 (CC3) staining in D425 orthotopic tumor sections 3 weeks after injection (tumor area encircled with black line; %‐positive cells indicated in each figure; scale bar on bottom left = 100 μm). (C) HDMB03 cell flank tumors harvested from NSG mice after six doses of intratumor‐injected vehicle (ethanol) or 1632 treatment. Scale with inch demarcation is shown for size comparison (also scale bar on bottom left of panel C = 1 inch). (D) Tumor volume change following 1632 treatment compared with vehicle control. Tumors were derived from either HDMB03 or D341 cells. Unpaired *t*‐test *P*‐value: **P < 0.05 (*n* = 10 for control; *n* = 11 for 1632). (E) Immunoblots of vehicle or 1632 treated HDMB03 xenograft flanks demonstrate reduction in LIN28B, PBK, c‐MYC KRAS, phospho‐Rb, and phospho‐histone H3 after 1632 treatment. Error bars represent SEM.

To examine the role of the LIN28B pathway on tumor growth *in vivo*, we injected the *LIN28B* knockdown and corresponding control Group 3 MB cells into the cerebella of immunocompromised (NSG) mouse pups (p3‐p5). All injected mice were euthanized due to symptoms of tumor burden between 4 and 8 weeks after injection. Mice injected with the *LIN28B* knockdown cells survived significantly longer (log‐rank test *P* < 0.0005) than mice injected with control Group 3 MB cells (Fig. 6A), indicating a role for LIN28B in promoting Group 3 MB growth *in vivo*. We analyzed tumors isolated from a subset of orthotopic xenografts before symptom onset and observed that *LIN28B* knockdown tumors had decreased Ki‐67 and increased cleaved caspase‐3 levels compared with control tumors with high LIN28B levels (Fig. 6B and Fig. S6a). In the animals that had to be euthanized due to tumor burden, we observed that the *LIN28B* knockdown tumors re‐expressed or overexpressed LIN28B (Fig. S6b) rather than using an alternate pathway to drive tumor growth.

To investigate whether we could pharmacologically inhibit LIN28B in Group 3 MB *in vivo*, we injected 4–6‐week‐old NSG mouse flanks with HDMB03 or D341 cells and treated intratumorally with 40 mg·kg^−1^ of 1632, three times a week. We used flank injections because whether 1632 crosses the blood–brain barrier has not been determined, and we used this dosing regimen based on a previously published study, wherein compound 1632 was used *in vivo* [30] We found a significant reduction in the rate of tumor growth (and in one case, an objective reduction in the tumor size) after only six doses (12 days) of treatment (Fig. 6C,D; Fig. S6c,d). Immunoblotting confirmed reduction in LIN28B levels as well as its downstream targets MYC, PBK, and KRAS, as well as pRb and pHH3 (Fig. 6E and Fig. S6e), supporting the hypothesis that the observed volume reduction is specifically due to the inhibition of LIN28B.

### 3.6 Pharmacologic inhibition of PBK leads to decreased viability and proliferation of Group 3 MB cells

**Fig. 7:**
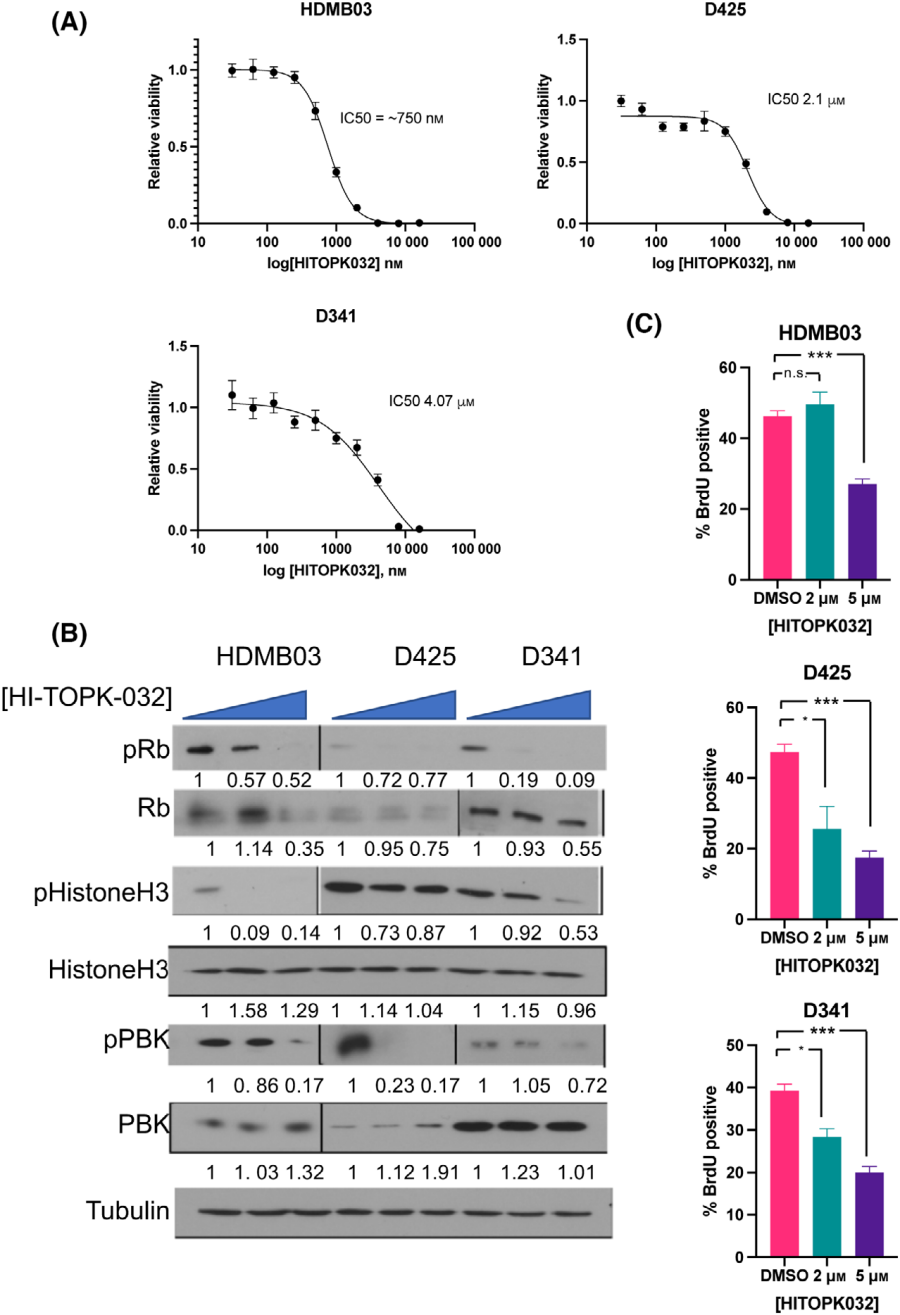
PBK inhibition with HI‐TOPK‐032 significantly decrease Group 3 medulloblastoma viability. (A) Increasing concentration of HI‐TOPK‐032 treatment decreases viability of Group 3 MB cells. The IC50 calculated for D341, D425, and HDMB03 cell lines (~ 750 nm to 2 μm) are demonstrated as well (representative image shown, experiment performed with three biological replicates; error bars represent SEM calculated from six technical replicates). (B) Treatment with increasing concentration (from left to right, DMSO, 2, 5 μm) of the PBK inhibitor HI‐TOPK‐032 leads to decrease in phospho‐PBK, phospho‐Rb, and phospho‐histone H3 (*n* = 3 per group unless otherwise stated; relative protein amounts calculated based on densitometry analysis shown below each blot). (C) BrdU incorporation analysis demonstrates dose‐dependent decrease in BrdU incorporation in D341, D425, and HDMB03 cells following treatment with HI‐TOPK‐032 (unpaired *t*‐test *P*‐value: *P < 0.05; ***P < 0.001; *n* = 3 per group). Error bars represent SEM.

Since knockdown of *PBK* resulted in decreased viability of Group 3 MB cells similar to knockdown of *LIN28B* (Fig. 3F), we wanted to determine whether pharmacologic inhibition of PBK would have similar effects. We treated Group 3 MB cell lines with increasing concentrations of HI‐TOPK‐032, a PBK inhibitor, and found a dose‐dependent reduction in cell viability (Fig. 7A). As shown in Fig. 7B, treatment with 2–5 μm HI‐TOPK‐032 leads to decreased levels of phospho–PBK, pRb, and pHH3, suggesting that reduced cell cycle progression is a mechanism for the efficacy of this drug in Group 3 MB. We also found that the IC50 for this agent ranged from 750 nm to 4 μm, which is much lower than that of compound 1632 (Fig. 4B). Finally, to investigate whether the reduction in viability was due to a decrease in proliferation, we performed BrdU incorporation analysis in Group 3 MB cells following treatment with HI‐TOPK‐032 and found a significant reduction in BrdU incorporation by immunofluorescence compared with vehicle alone (Fig. 7C and Fig. S7).

## 4 Discussion

Patients with Group 3 MB have a poor prognosis with a 5‐year survival rate of less than 60%. The cancer dependency map project catalogs genome‐wide genetic vulnerabilities through RNAi and CRISPR‐Cas9 whole‐genome loss of function screens performed on over 600 cancer cell lines [23, 24, 25]. These studies suggest that Group 3 MB cells appear to require LIN28B for viability as compared to other RNA‐binding proteins (RBPs) (CERES score range −0.32 to −0.579; *P*‐value 1.0 e‐05) and when compared to other cancer cell lines. Thus, targeting LIN28B and pathways regulated by LIN28B could be an effective strategy to treat Group 3 MB.

In this study, we demonstrate LIN28B's essential role for Group 3 medulloblastoma cell proliferation and reveal the LIN28B–let‐7–PBK axis as a major pathway exploited by these cancer cells. *PBK* was recently identified as a hub gene in medulloblastoma by Deng et al. [15]. Here, we demonstrate that this stems from repression of let‐7i due to overexpression of *LIN28B*. Finally, we demonstrate that pharmacologic agents targeting LIN28 and PBK reduce viability of Group 3 cells and *in vivo* tumor growth.

A role for LIN28B in Group 3 medulloblastoma was suggested by genome‐wide methylation analysis by Hovesteadt et al. [4] demonstrating hypomethylation of the LIN28B locus, correlating with high *LIN28B* and low *let‐7* expression along with survival data demonstrating poor outcome in patients with high *LIN28B* expression ([4] and our own analysis). While Rodini et al. [32] did not see a significant difference in prognosis for patients with high LIN28 expression, this is most likely due to the fact that the authors focused only on LIN28A, which appears to be expressed at low levels across medulloblastoma patient samples, rather than LIN28B. Our observation of a significant survival difference between patients with high and low *LIN28B* expression may be in part explained by the difference in *MYC* expression. Several prior studies have demonstrated *LIN28B* is transcriptionally regulated by MYC [6]. We also found that downregulation of LIN28B led to decreased levels of MYC, supporting the positive feedback loop between LIN28B and MYC.

More recently, Maklad et al. [5] showed that LIN28B is a prognostic marker in medulloblastoma and that compound 1632 is effective in decreasing expression of *LIN28B* and growth of medulloblastoma cell lines. Our findings are consistent with these, but in addition, we have done shRNA‐mediated knockdown studies which add confidence that these effects indeed are specific to LIN28B. We also demonstrate that this growth difference is primarily due to difference in cell proliferation and that this may be due to regulation of PBK via let‐7. In addition, we have demonstrated *in vivo* survival differences in orthotopic models as well as the efficacy of using compound 1632 in flank models. We have also investigated an inhibitor of PBK and demonstrated *in vitro* efficacy in reducing Group 3 MB cell proliferation.

LIN28B inhibits maturation of the microRNA let‐7 miRNA, which in turn down‐regulates the expression of *LIN28B* and other oncogenes including *PBK*, *MYC*, *IGFBP2*, *HMGA2*, and *KRAS* [6, 28]. LIN28B was previously shown to act through the let‐7–PBK axis by Chen et al. [7] in neuroblastoma, another common small round blue cell tumor of childhood. LIN28B can also regulate expression of genes in a let‐7 independent fashion [6, 33, 34, 35] and may regulate *PBK* in such a manner as well, although that mechanism has not been described to date. *PBK* is also regulated by other transcription factors [36] although whether this mechanism is active in Group 3 MB is not known. While we demonstrate that inhibition of PBK decreases viability in a manner similar to LIN28B inhibition, it is possible that these proteins are acting independent of each other. In our study, the let‐7–PBK axis appears to be an important mechanism of LIN28B regulation of PBK and other downstream targets in Group 3 MB‐although we did not investigate other pathways which may contribute to this interaction between LIN28B and PBK. We specifically investigated let‐7i since that was the miRNA demonstrated to inhibit PBK by Chen et al. [7]; it is possible other let‐7 miRNAs are also acting in a similar fashion.

LIN28A and LIN28B are well‐known regulators of cell fate determination and self‐renewal. Wefers et al. [37] investigated the ability of LIN28B to initiate CNS tumor development *in vivo* in mice. In their study, overexpression of Lin28b alone was not sufficient for tumor formation in Math1‐positive cerebellar granular neuron precursors or Nestin‐positive neuronal precursor cells. Rather, LIN28B overexpression led to hypersublobulation of the cerebellar vermis. Overexpression of LIN28A alone also appears to be insufficient to form CNS tumors [38]. However, in our study, we found LIN28B overexpression increased tumor sphere formation in a Group 3 MB cell line. This suggests that while LIN28B may not alone be sufficient for tumor establishment, it does play an important role in cancer growth. Recently, Smith et al. [39] demonstrated that Group 3 tumors arise from human fetal rhombic lip cells, which are distinct from cerebellar granular neuron precursors; thus, Wefers et al. may have underestimated the contribution of LIN28B in Group 3 MB pathogenesis.

None of the LIN28 inhibitors developed to date have demonstrated sufficient preclinical efficacy to advance to clinical studies. We tested one LIN28 inhibitor, 1632, which was demonstrated to have *in vivo* preclinical efficacy in *LIN28B*‐overexpressing subtypes of Ewing sarcoma and in oral squamous cell carcinoma [29, 30]. Our studies demonstrated that treatment of Group 3 MB cells with 1632 is able to reduce cell cycle progression by inhibiting LIN28B and its downstream targets. The effect of 1632 on Group 3 MB cell viability is significantly abrogated when we knockdown *LIN28B* (Fig. S5b), suggesting that this drug is specific to LIN28. However, in our studies, the IC50 for 1632 was ~ 150 μm (range 126–169 μm), and while that is similar to the range seen in Ewing sarcoma cells, it is unclear whether these levels are achievable or tolerable in the CNS. Based on our limited studies, these doses should be tolerated by normal astrocytes and the CNS cells surrounding the tumor (Fig. 5). In addition, our ability to demonstrate its efficacy *in vivo* even in heterotopic models is encouraging and warrants further investigation.

Given LIN28B is a stem cell factor, it is possible that by inhibiting LIN28B, in addition to its inhibitory effect on cell cycle progression, 1632 leads to differentiation of Group 3 MB cells. While we did not specifically test this hypothesis, we found that the levels of the stem cell factor SOX2 and expression of CD133 are decreased following 1632 treatment. However, the stem cell factor Oct4 appears to increase, while Nanog appears unchanged (data not shown). Thus, we cannot make any conclusions about the effect of 1632 on differentiation from our limited studies and future studies will need to address this important question.

We also investigated the PBK inhibitor HI‐TOPK‐032, which was recently demonstrated by Deng et al. [15] to decrease MB cell proliferation in D341 (Group 3) and DAOY (SHH) cell lines. In our experiments, we found HI‐TOPK‐032 to decrease cell viability in D341, D425, and HDMB03 Group 3 MB cell lines at relatively low IC50s. In contrast to Deng et al., we used shRNA to knockdown *PBK* expression to demonstrate the specific effect of PBK loss in Group 3 MB, both in MB cell lines (D341 and HDMB03) and in a patient‐derived primary cell (BT52). This complements the study done by Deng et al. and adds to the evidence that PBK is a major regulator in MB, due to upstream overexpression of LIN28B. Furthermore, PBK inhibition has been studied as a strategy to treat many cancers including brain tumors [40]. As kinase inhibition is a well‐known strategy in oncology [41], and potentially easier than inhibiting an RNA‐binding protein, PBK inhibition may be the most effective strategy to target the LIN28B pathway.

## 5 Conclusion

Taken together, our data support the hypothesis that LIN28B and its downstream target PBK are important regulators of Group 3 medulloblastoma cell proliferation and that targeting the LIN28B–PBK axis with pharmacologic therapy is plausible. In contrast to previous studies, we have used knockdown and overexpression studies to demonstrate the specific role of LIN28B and PBK in addition to pharmacotherapies. Our work adds to the growing body of literature supporting the crucial role of LIN28B in the development of tumors. Given the discovery by Hovestadt et al. [4] that the *LIN28B* promoter region is hypomethylated in Group 3 MB, we did not explore additional mechanisms for *LIN28B* upregulation in Group 3 MB. However, c‐Myc is frequently upregulated in Group 3 MB and has been implicated as a possible transcriptional activator of *LIN28B*. When we compared the single‐cell gene expression map of *LIN28B* and *MYC*, we did not notice a direct correlation, although this does not exclude the possibility of post‐transcriptional regulation. Additional studies will help further evaluate the cause of *LIN28B* upregulation in medulloblastoma.

The pharmacologic agents 1632 and HI‐TOPK‐032 have demonstrated efficacy in preclinical studies but neither have been tested in clinical studies as yet. HI‐TOPK‐032 has been tested in a glioma model using heterotopic xenografts, not orthotopic models. Thus, we have no information on whether either agent is able to traverse the BBB yet. We are currently investigating the ability of these agents to cross the BBB and additional *in vivo* experiments to evaluate the efficacy of these drugs in animal models as well as potential combination strategies. Future studies will characterize the *in vivo* dosages and efficacy of these agents before proceeding to potential clinical trials. We are also exploring additional roles of LIN28B including in regulating metastasis as well as the roles of its other downstream targets in medulloblastoma. These studies will lay the groundwork for helping us understand the role of this very important pathway and allow better therapeutic options for patients with medulloblastoma and other cancers where this pathway is dysregulated.

## Conflict of interest

The authors declare no conflict of interest.

## Author contributions

SWS, CMR, JS, HK, and LFM performed all the experiments. KJ, SAK, and OS performed computational analysis of RNA‐Seq data and generated figures. MDT provided tissue for RNA‐Seq and funding support for analysis. MS provided tissue sections for immunohistochemistry. SWS, RWS, TJM, and AMK conceptualized the study. SWS analyzed and interpreted the data. SWS wrote the manuscript and generated figures. All authors read and approved the final manuscript.

### Peer review

The peer review history for this article is available at https://www.webofscience.com/api/gateway/wos/peer‐review/10.1002/1878‐0261.13477.

## Supporting information


**Fig. S1.** *LIN28A* and *MYC* expression in MB patients and cells.
**Fig. S2.** *LIN28B* qPCR, shLIN28B‐5 knockdown, BT52 viability, and D556 *LIN28B* overexpression.
**Fig. S3.** Immunofluorescence images of *LIN28B* knockdown.
**Fig. S4.** LIN28B–let‐7–PBK axis in D341, D425, and BT52 cells.
**Fig. S5.** Effect of 1632 on *LIN28B* knockdown cells.
**Fig. S6.** *In vivo* effect of *LIN28B* knockdown and 1632 treatment.
**Fig. S7.** Immunofluorescence images of HI‐TOPK‐032 treatment.
**Table S1.** List of primers for miRNA qPCR.
**Table S2.** Cell cycle analysis of BrdU incorporation following 1632 treatment.

## Data Availability

No new data were generated in this study. The RNA‐Seq data that support the findings of this study are obtained from the previously published studies accessible at https://doi.org/10.1038/s41467‐021‐21883‐0 and at https://doi.org/10.1038/s41586‐022‐05215‐w and have been deposited in the European Genome‐Phenome Archive (EGA) database under the accession codes EGAD00001006305 and EGAS00001005826. The RNA‐Seq data on the WNT subgroup patients are not yet publicly available due to pending publication (but will be made available as soon as accepted for publication). The microarray data used for performing survival analysis are also previously published (https://doi.org/10.1016/j.ccell.2017.05.005) and available in GEO under accession GSE85218.
