# Supplementary material for: The LIN28B–let‐7–PBK pathway is essential for group 3 medulloblastoma tumor growth and survival
Source: Mol Oncol. 2023 Aug 7;17(9):1784–802. doi: 10.1002/1878-0261.13477 (PMC10483609; doi:10.1002/1878-0261.13477)

Supplemental Figure S1. (a) LIN28A expression patterns in patients with different subgroups of MB based on bulk RNA-seq data (25th to 75th percentile marked with box limits while whiskers denote 1.5x interquartile range beyond 25th and 75th percentile). (b) UMAP plot of single cell transcriptome analysis from 28 MB patients (1 WNT, 9 SHH, 7 GP3 and 11 GP4) with WNT in yellow, SHH in blue, Group 3 in green and Group 4 in orange (numbers refer to UMAP subclusters; Subcluster 2, 5, 8, 9, 10, 15 as well as 3 [partial] and 11 [partial] belong to Group 3). Relative expression of LIN28B (gradient of expression from light yellow to dark brown, blue = no expression) in all MB patients and MYC RNA expression in Group 3 patients are displayed for comparison. (c) in the SHH subgroup of medulloblastoma, there is no significant difference in LIN28B expression between P53 wild type versus mutant tumors (25th to 75th percentile marked with box limits while whiskers denote 1.5x interquartile range beyond 25th and 75th percentile). (d) cMYC (positive control) staining of tissue sections from Group 3 MB patients (e) Western blot of Group 3 MB cell lines D341, D425, D556, LIN28A+ AT/RT cell line CHLA06 and SHH cell line ONS76 demonstrating low levels of LIN28A across medulloblastoma cells.

Supplemental Figure S2. (a) Quantitative real-time PCR analysis in D341, D425 and HDMB03 cells demonstrate relative LIN28B expression is decreased in shRNA treated cells compared to control. TBP is used as reference gene (b) shRNA knockdown of LIN28B levels following treatment of D425, D341 and HDMB03 cells with shLIN28B-5 compared to controls (shctl). (c) shRNA knockdown of LIN28B levels in BT52 primary patient cells lead to decreased cell viability. (d) BrdU incorporation analysis in Group 3 MB cell lines following LIN28B knockdown using shLIN28B-5 demonstrates decreased BrdU incorporation in knockdown cells compared to control. (e) LIN28B overexpression in D556 cells also leads to increase in viability and significant increase in the number of tumor spheres. Representative images of tumor sphere formation assay using D556 cells transfected with either control vector LV105 or LIN28B

overexpression plasmid (LVLIN28B). (Error bars represent SEM; Unpaired t-test p-value: * $p < 0.05$; ** $p < 0.01$; *** $p < 0.001$; **** $p < 0.0001$; $n = 3$ per group)

Supplemental Figure S3. Representative images demonstrating BrdU incorporation of D341 cells treated with either control plasmid (shctl) or LIN28B knockdown plasmid (shLIN28B-1 and shLIN28B-5). Scale bar shown in bottom left = 120 μm.

Supplemental Figure S4. (a) LIN28B knockdown in D425 cells leads to increased let-7i and let-7g expression in the shLIN28B treated cells compared to controls. (b) Concurrently we observed decreased levels of PBK and HMGA2 protein compared to cells treated with control shRNA (shctl).(c) Real-time quantitative PCR demonstrates significant upregulation of let-7i expression following transfection.(d) Immunoblots following transient transfection of let-7i in D341, D425 and HDMB03 cell lines demonstrate reduced levels of LIN28B, PBK and KRAS compared to negative control (miRNC) (densitometric analysis demonstrated under each blot). (e) Decreased viability of BT52 patient cells treated with shRNAs targeting PBK compared to control. Western blot showing PBK knockdown in BT52 cells shown below. (Error bars represent SEM; Unpaired t-test p-value: * $p < 0.05$; ** $p < 0.01$; *** $p < 0.001$; **** $p < 0.0001$; $n = 3$ per group)

Supplemental Figure S5. (a) Immunoblots demonstrate LIN28B levels in D341 and D425 cells following treatment with shRNA (shLIN28B-1 or shLIN28B-5). (b) In cells with very notable LIN28B knockdown as with shLIN28B-1 (middle), viability is not significantly reduced after 250 μM 1632 treatment compared to DMSO control. However, for cells with less significant knockdown as with shLIN28B-5 (right), 1632 treatment appears to still reduce viability compared to vehicle control. (Error bars represent SEM; Unpaired t-test p-value: * $p < 0.05$; ** $p < 0.01$; *** $p < 0.001$; **** $p < 0.0001$)

Supplemental Figure S6. (a) Immunohistochemistry demonstrating LIN28B, Ki-67, Cleaved Caspase 3 (CC3) and MYC staining in D341 orthotopic tumor sections 3 weeks after orthotopic injection. As demonstrated in the figure, Ki-67 decreases from 79.1% in the control to 61.8% in the knockdown, and cleaved caspase 3 increases from 41% to 72.7%) (b) Levels of LIN28B in orthotopic tumor samples at the time of death. These tumors were generated from orthotopic injection of D341 cells transfected with either shLIN28B-1, shLIN28B-5 or control shRNA (shctl). (c) D341 flank tumors after 5 doses of vehicle control or 40 mg/kg every other day 1632 intratumoral injection. (d) Volume change of D341 or HDMB03 flank tumors after 10 days (5 treatments) (Error bars represent SEM; Unpaired t-test p-value: * $p < 0.05$) (e) Immunoblot demonstrating decrease in LIN28B, PBK, phospho-Rb, phospho-Histone H3 in lysates from D341 flank tumors following 1632 treatment compared to vehicle control.

Supplemental Figure S7. Representative images of BrdU staining of HDMB03, D425 and D341 cells treated with DMSO, 2 μM and 5 μM HI-TOPK-032. Scale bar at bottom left = 100 μm.

Supplemental Table S1. List of qPCR primers for miRNA qPCR.

Sequence Name	Sequence
Universal Taqman Probe	/56-FAM/CAG AGC CAC /ZEN/CTG GGC AAT
let7g_RT	CAG TGC AGG GTC CGA GGT CAG AGC CAC CTG
let7g_F	TCG GTG AGG TAG TAG TTT GTA
let7i_RT	CAG TGC AGG GTC CGA GGT CAG AGC CAC CTG GGC AAT TTT TTT TTT TAA CAG C
let7i_F	CGG TGA GGT AGT AGT TTG TG
Universal Reverse Primer	CAG TGC AGG GTC CGA GGT
RNU44_RT	CAG TGC AGG GTC CGA GGT CAG AGC CAC CTG GGC AAT TTT TTT TTT TTT AGT C
RNU44_F	TGG CCT GGA TGA TGA TAA GCA

Supplemental Table S2. Representative fraction (%) of cells in each phase of cell cycle (Sub G0, G0/G1, S and G2/M, following treatment with DMSO or 50, 150 or 250 µM 1632. Two-tailed paired t-test p-value for cells in S phase comparing each drug dose to DMSO control is listed in parenthesis (based on 3 separate biological replicates including this one).

D341	DMSO	50 µM	150 µM	250 µM
**Sub-G0**	31.2	34.9	36.7	40.8
**G0/G1**	34.4	39.5	41.9	35.1
**S**	23.4	22 (n.s.)	16.6 (0.0058)	11.6 (0.0262)
**G2/M**	8.99	2.6	3.73	10.1

HDMB03	DMSO	50 µM	150 µM	250 µM
**Sub-G0**	25.7	30.7	39.5	42.9

**G0/G1**	40.6	40	40.1	42.3
**S**	32.3	28.4 (n.s)	19.4 (0.0273)	12 (0.0352)
**G2/M**	0.021	0.029	0.057	1.09

**D425**	**DMSO**	**50 µM**	**150 µM**	**250 µM**
**Sub-G0**	19.6	30.3	29.5	46.2
**G0/G1**	42.2	39.1	44.4	34.1
**S**	30	20.4 (0.0416)	16.2 (0.0106)	9.29 (0.0389)
**G2/M**	6.22	9.13	9.22	10.3

Shahab Figure S1

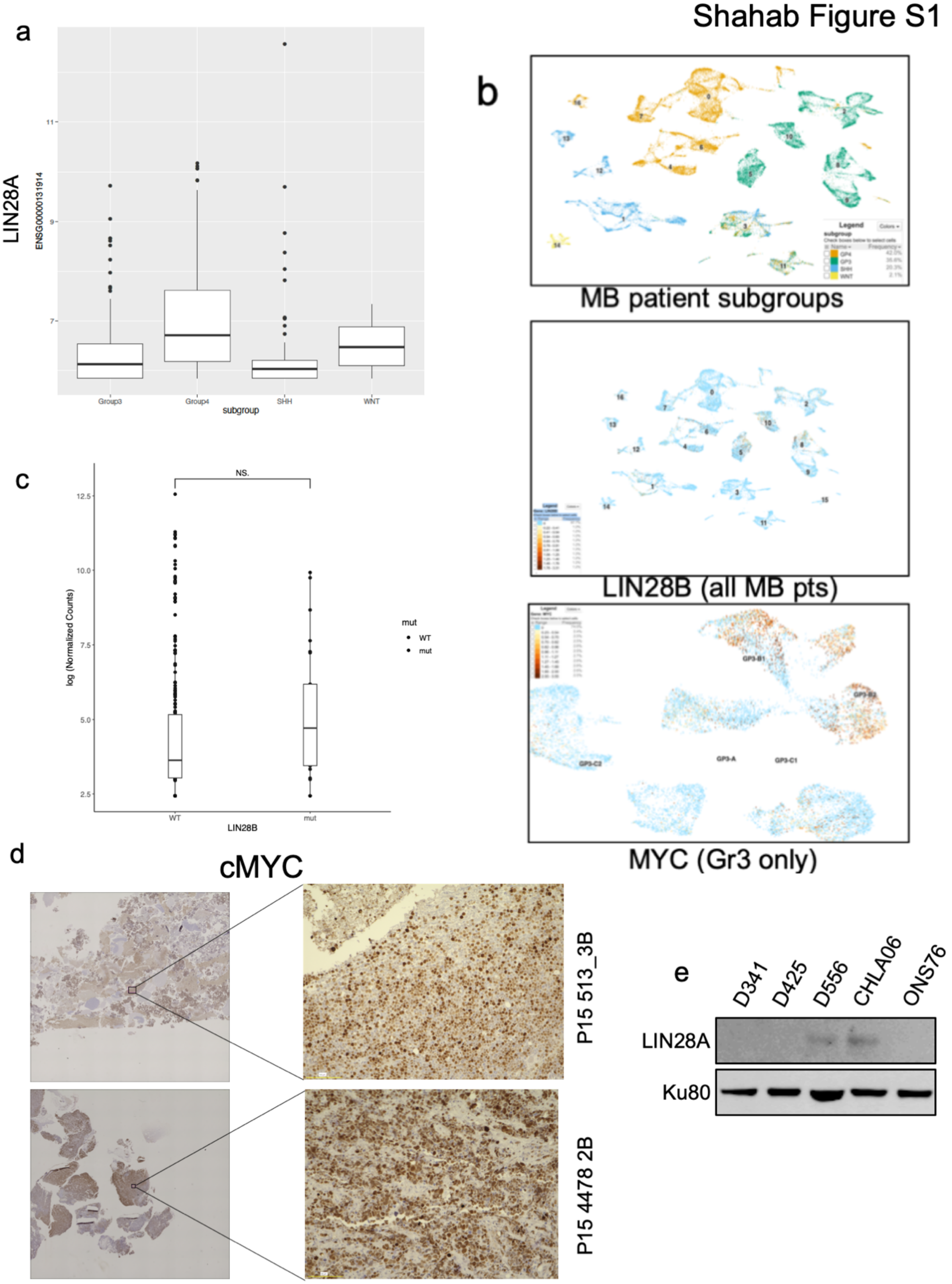

Shahab Figure S2

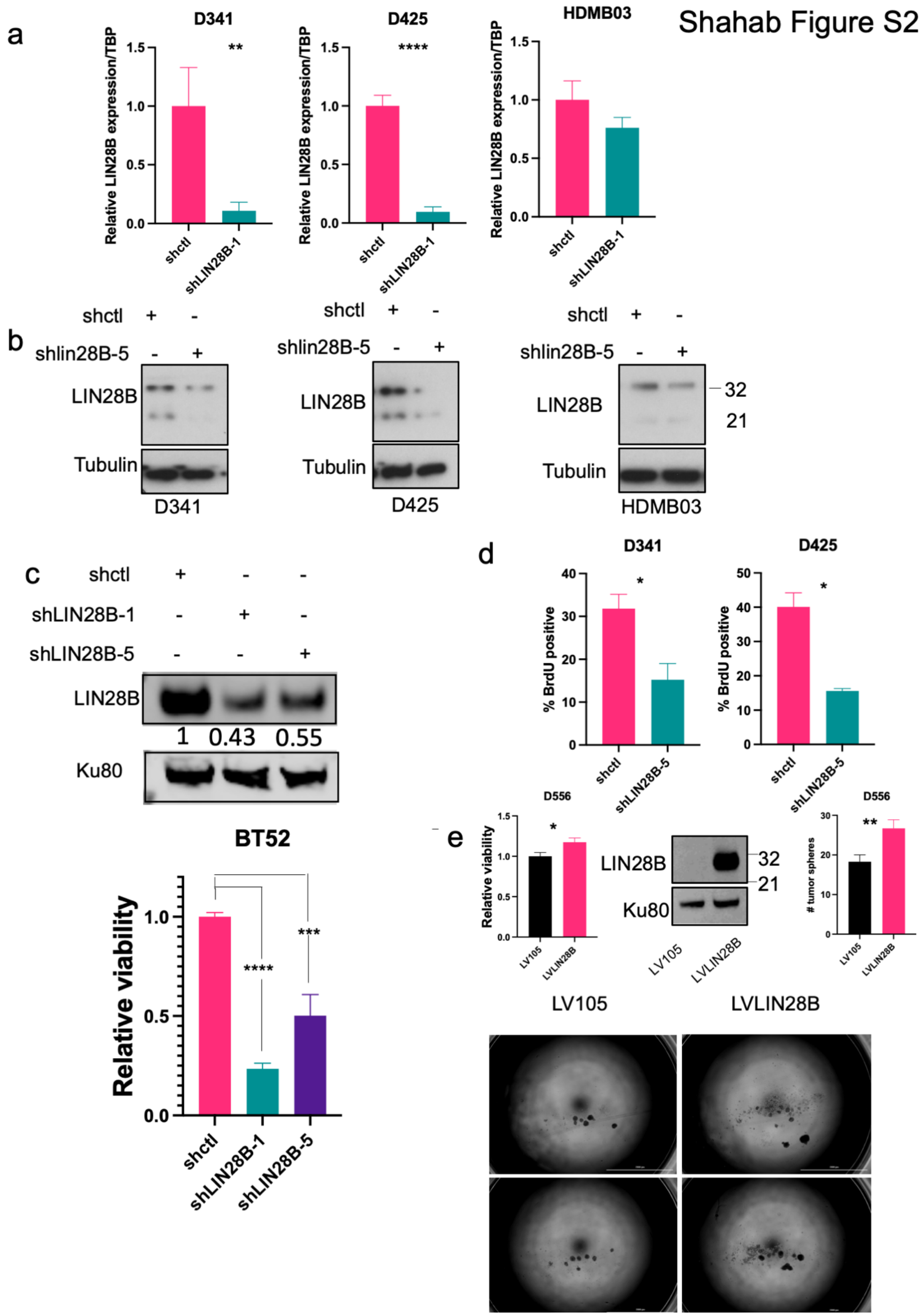

Shahab Figure S3

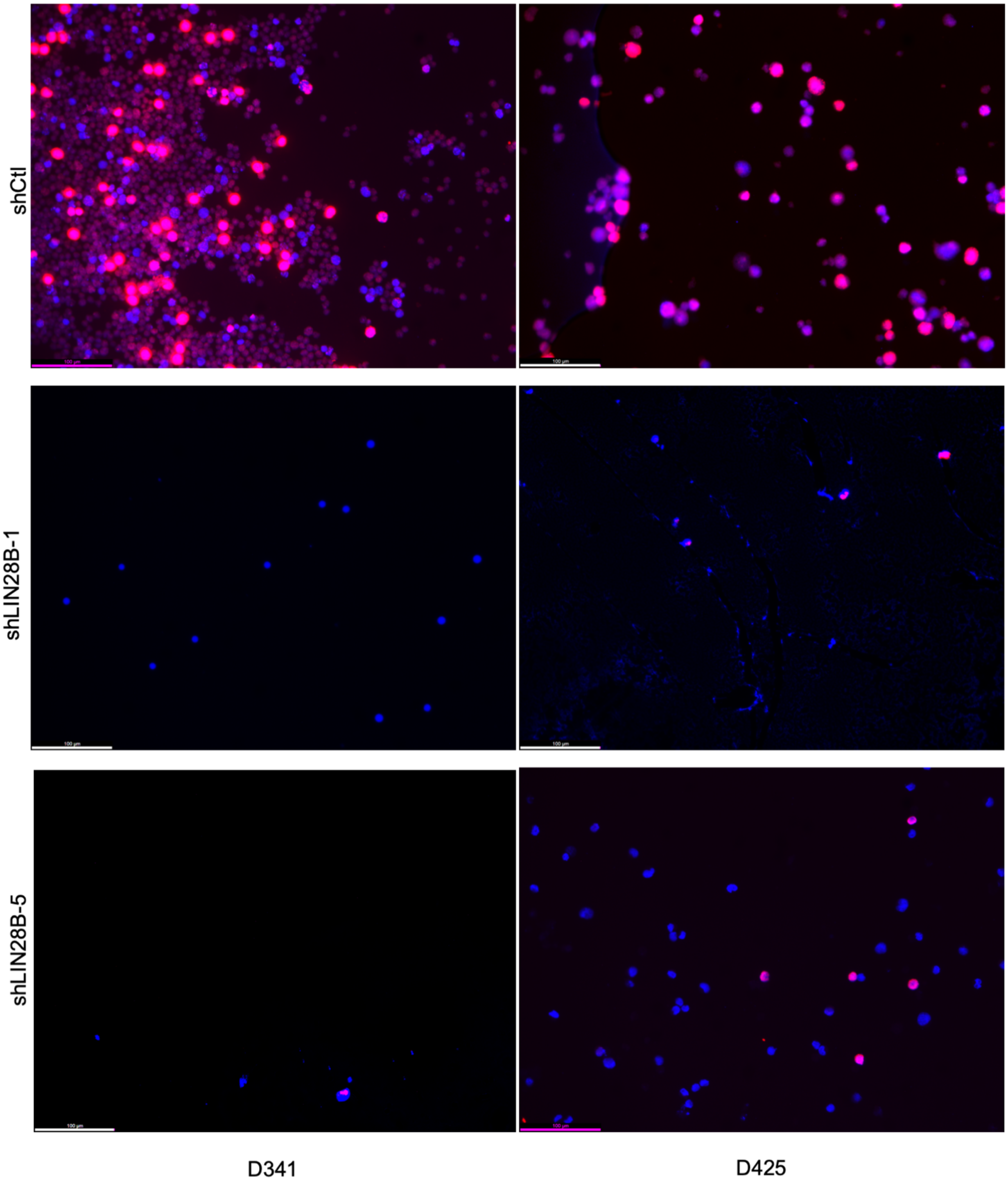

Shahab Figure S4

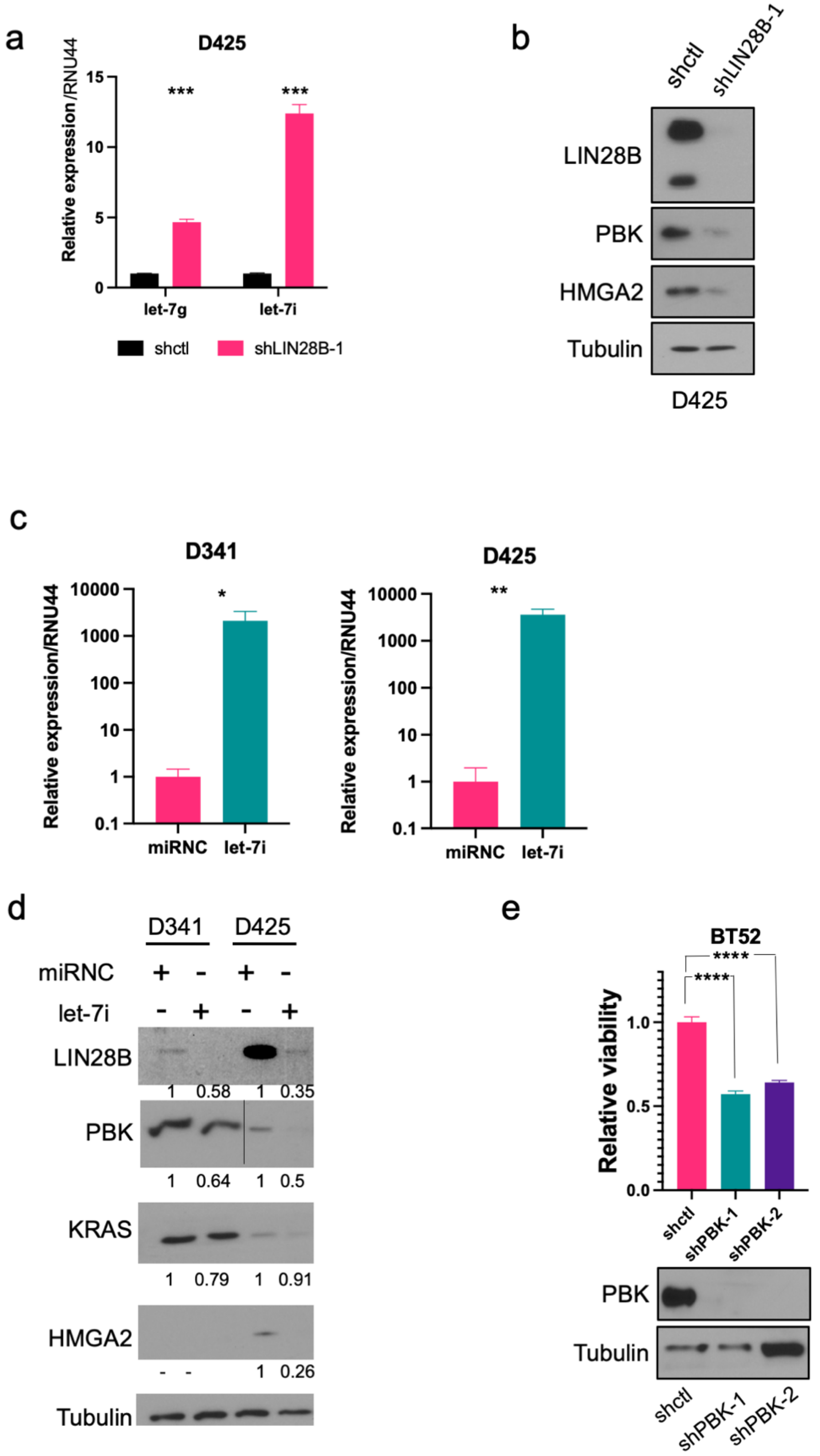

Shahab Figure S5

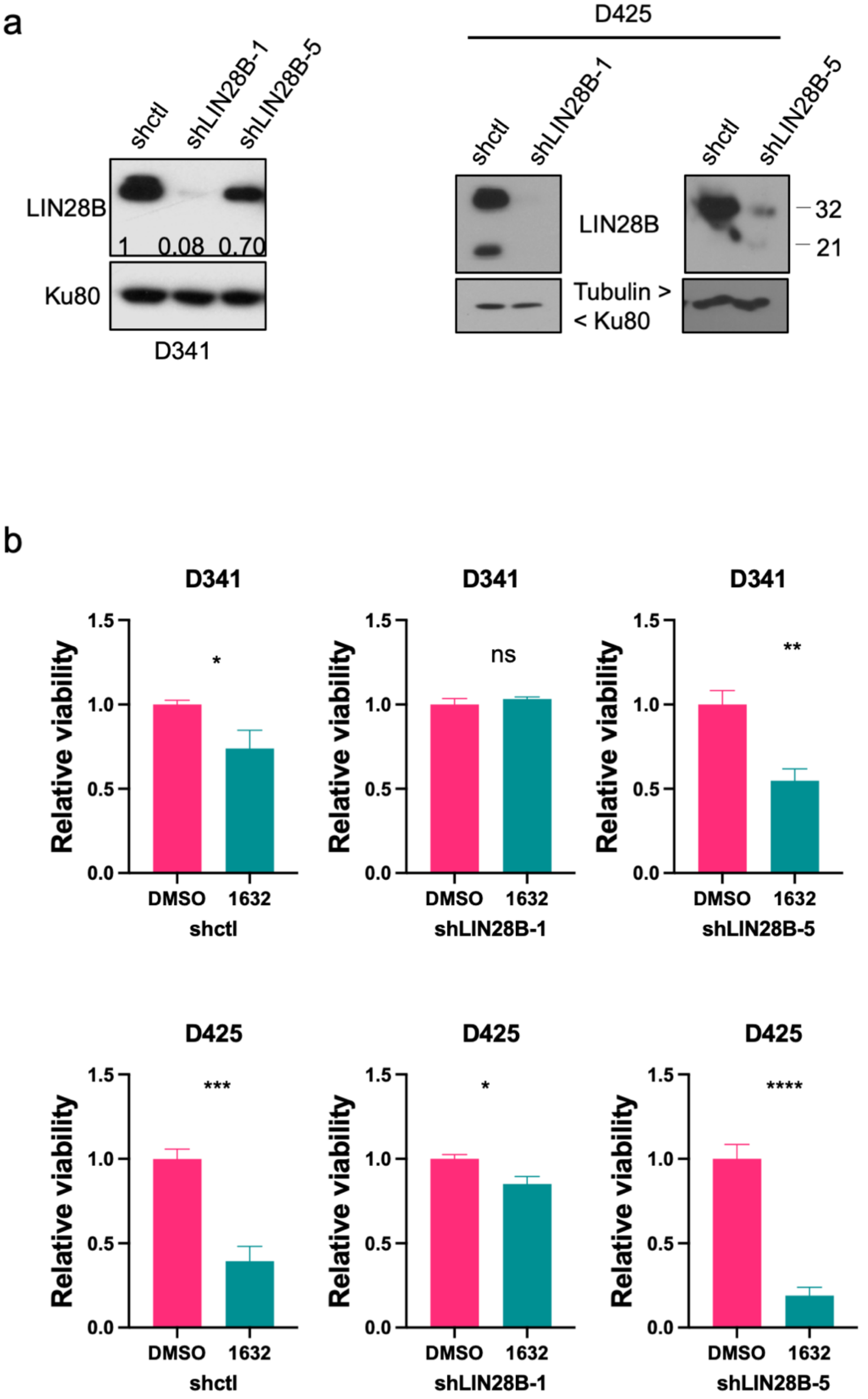

Shahab Figure S6

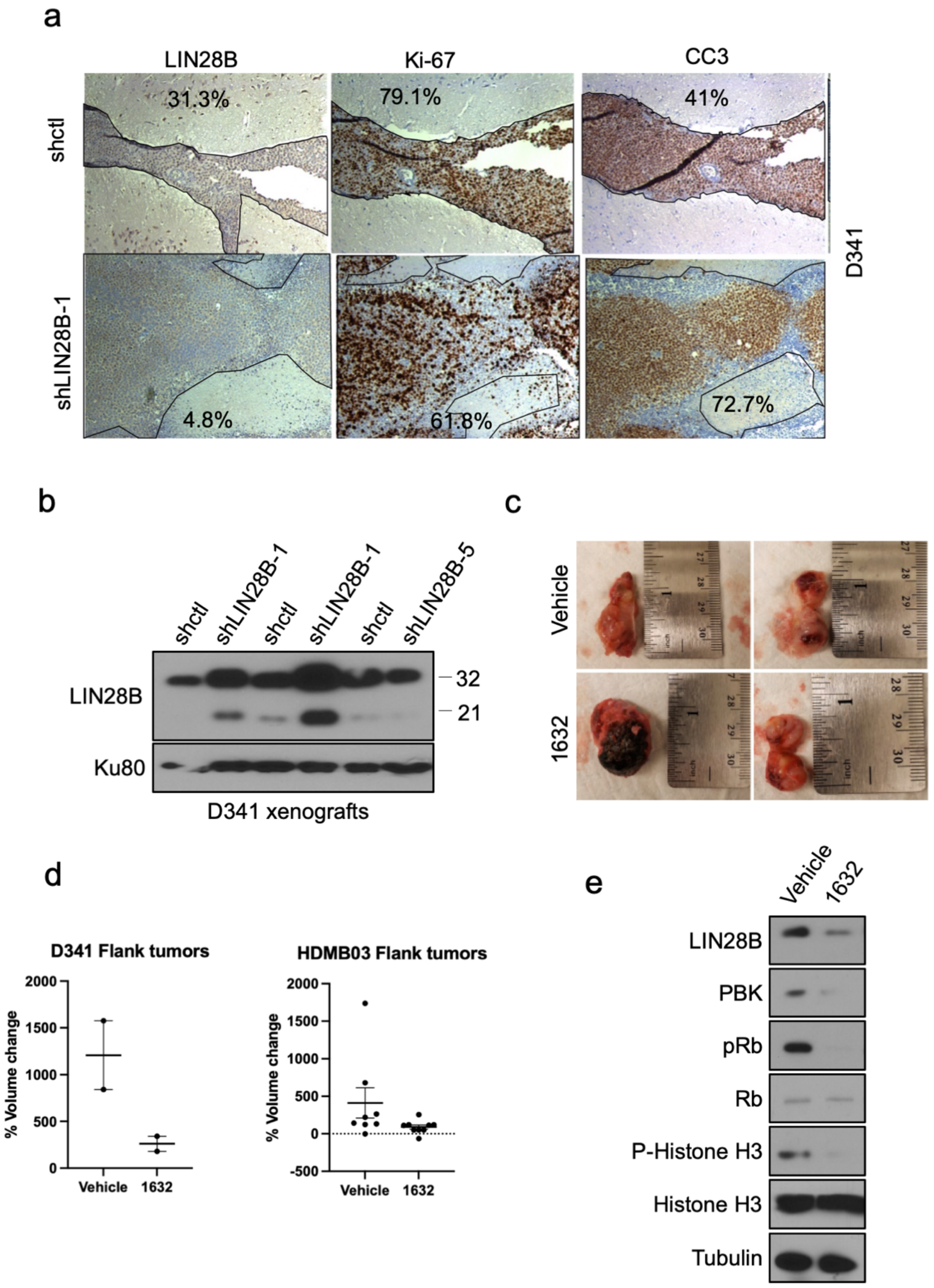

Shahab Figure S7

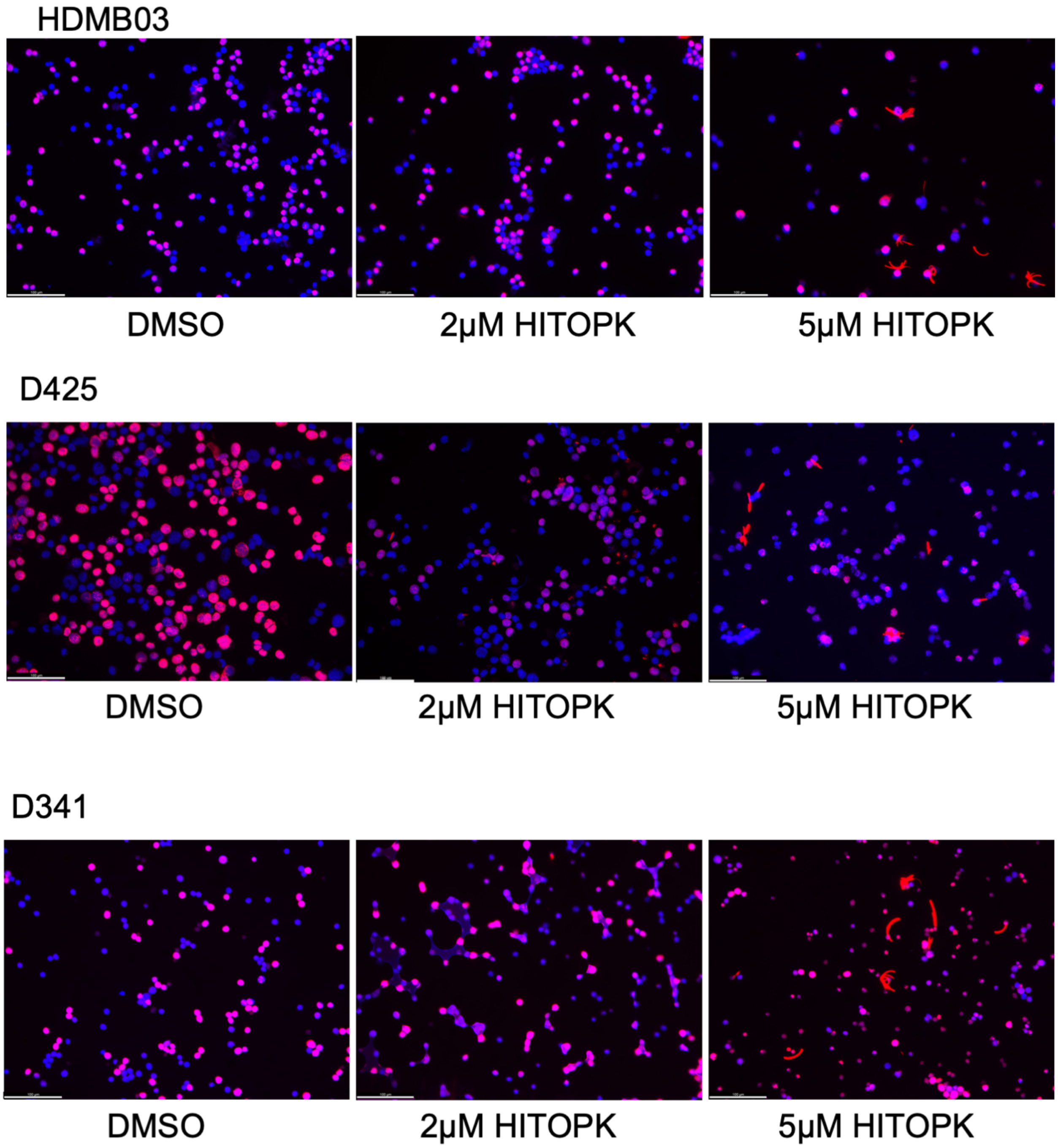

Supplement: Supplementary file 1 — Fig. S1. LIN28A and MYC expression in MB patients and cells. Fig. S2. LIN28B qPCR, shLIN28B‐5 knockdown, BT52 viability, and D556 LIN28B overexpression. Fig. S3. Immunofluorescence images of LIN28B knockdown. Fig. S4. LIN28B–let‐7–PBK axis in D341, D425, and BT52 cells. Fig. S5. Effect of 1632 on LIN28B knockdown cells. Fig. S6. In vivo effect of LIN28B knockdown and 1632 treatment. Fig. S7. Immunofluorescence images of HI‐TOPK‐032 treatment. Table S1. List of primers for miRNA qPCR. Table S2. Cell cycle analysis of BrdU incorporation following 1632 treatment. [file MOL2-17-1784-s001.pdf]
